# Exposure to elevated sea-surface temperatures below the bleaching threshold impairs coral recovery and regeneration following injury

**DOI:** 10.7717/peerj.3719

**Published:** 2017-08-18

**Authors:** Joshua Louis Bonesso, William Leggat, Tracy Danielle Ainsworth

**Affiliations:** 1 College of Public Health, Medical and Veterinary Sciences, James Cook University, Townsville, Australia; 2 ARC Centre of Excellence for Coral Reef Studies, James Cook University, Townsville, Australia

**Keywords:** Coral reefs, GFP, Heat shock proteins (HSP), Injury, Recovery, Scanning Electron Microscopy (SEM), Temperature stress

## Abstract

Elevated sea surface temperatures (SSTs) are linked to an increase in the frequency and severity of bleaching events due to temperatures exceeding corals’ upper thermal limits. The temperatures at which a breakdown of the coral-*Symbiodinium* endosymbiosis (coral bleaching) occurs are referred to as the upper thermal limits for the coral species. This breakdown of the endosymbiosis results in a reduction of corals’ nutritional uptake, growth, and tissue integrity. Periods of elevated sea surface temperature, thermal stress and coral bleaching are also linked to increased disease susceptibility and an increased frequency of storms which cause injury and physical damage to corals. Herein we aimed to determine the capacity of corals to regenerate and recover from injuries (removal of apical tips) sustained during periods of elevated sea surface temperatures which result in coral stress responses, but which do not result in coral bleaching (i.e., sub-bleaching thermal stress events). In this study, exposure of the species *Acropora aspera* to an elevated SST of 32 °C (2 °C below the bleaching threshold, 34 °C) was found to result in reduced fluorescence of green fluorescent protein (GFP), reduced skeletal calcification and a lack of branch regrowth at the site of injury, compared to corals maintained under ambient SST conditions (26 °C). Corals maintained under normal, ambient, sea surface temperatures expressed high GFP fluorescence at the injury site, underwent a rapid regeneration of the coral branch apical tip within 12 days of sustaining injury, and showed extensive regrowth of the coral skeleton. Taken together, our results have demonstrated that periods of sustained increased sea surface temperatures, below the corals’ bleaching threshold but above long-term summertime averages, impair coral recovery from damage, regardless of the onset or occurrence of coral bleaching.

## Introduction

Sea surface temperatures (SSTs) have increased in recent decades and are projected to continue to increase at a rate of, on average, 0.12 °C per decade (Steig et al., 2009). Elevated SSTs have been shown to severely impede calcification and skeletal deposition in reef-building corals (Carricart-Ganivet et al., 2012). Synergistic stressors on coral reefs during periods of increased SSTs are also projected to become increasingly common. For example, storm and disease events are projected to increase in frequency and intensity (Hoegh-Guldberg et al., 2007; Hughes et al., 2003) and predation by crown of thorns starfish is predicted to increase in areas with decreasing water quality (De’arth et al., 2012; Brodie et al., 2005). These events in isolation, and where occurring together, will result in a far greater frequency of stress and injury to reef-building corals. However, the capacity of corals to survive and recover is dependent on their capacity to successfully repair and/or regenerate lost tissue and re-grow damaged branches (Kramarsky-Winter & Loya, 2000). This reformation of both skeleton and tissue at lesion sites is essential for colony survival, growth, reproduction and the prevention of disease (Oren, Benayahu & Loya, 1997; Van de Water et al., 2015a; Van de Water et al., 2015b).

Corals produce fluorescent proteins (FPs) (Dove, Hoegh-Guldberg & Ranganathan, 2001) which play integral roles in photoprotection (Salih et al., 2000), photosynthetic enhancement in low-light environments, antioxidant enhancement (Bou-Abdallah, Chasteen & Lesser, 2006; Palmer, Modi & Mydlarz, 2009), camouflage (Matz, Marshall & Vorobyev, 2006), regulation of the coral-*Symbiodinium* symbiosis (Dove et al., 2008; Field et al., 2006) and the coral innate immune response (Palmer, Modi & Mydlarz, 2009; D’Angelo et al., 2012). A principle role of Green Fluorescent Proteins (GFPs), photoprotection, regulates the light environment of the coral host tissue and protects the photosynthetic machinery of their endosymbionts from high irradiance (Salih et al., 2000; Schlichter, Weber & Fricke, 1985). In reef-building corals, GFP concentration has been found to change reversibly with light intensity, increasing in high-light corals and decreasing in medium to low-light corals (Roth et al., 2010). In fact, GFP concentration has been correlated with growth rates and light exposure, with high-light corals exhibiting faster growth rates and fluorescence than low light corals (Roth et al., 2010; Roth et al., 2010). Under high light conditions, GFPs dissipate excess energy and reflect light (Salih et al., 2000). Increased expression of GFP-like proteins can be found around corals’ growth zones where symbiont density is lowest, including the apical polyps, upper radial corallites, edges of healthy coral colonies and in areas of high or direct light exposure (D’Angelo et al., 2012). Here, GFPs have been hypothesised to reflect and scatter harmful and potentially damaging energy away from the endosymbionts (Salih et al., 2000; Schlichter, Weber & Fricke, 1985). Coral tissue compromised by injury, infection and the increased abundance of Reactive Oxygen Species (ROS) have altered GFP fluorescence and expression (Palmer, Modi & Mydlarz, 2009). For example, an up-regulation of GFP at the site of injured or compromised tissue functions as an antioxidant and scavenger of harmful oxygen radicals (e.g., H_2_O_2_, O${}_{2}^{-}$ and OH^−^) present at the wound site (Palmer, Modi & Mydlarz, 2009; Palmer, Roth & Gates, 2009).

Furthermore, specific GFP-like proteins and proliferating cell nuclear antigen growth markers have been found to show upregulation in growing branches and disturbed parts of colonies. This has been found to be important in the immune and repair response of reef-building corals following injury (D’Angelo et al., 2012). Studies have also shown a decrease in both concentration and expression of GFPs when corals are exposed to thermal stress and undergo bleaching (Dove, 2004; Smith-Keune & Dove, 2008; Desalvo et al., 2008). Under experimental conditions, rapid reduction in GFP concentration has been reported in *Montipora monastriata*, *Acropora yongei*, *Acropora millepora* and *Acropora aspera* after exposure to thermal stress (Smith-Keune & Dove, 2008) and a result of decreased symbiont densities (Roth & Deheyn, 2013). As a result, thermal stress has been postulated to overwhelm the photo-protective mechanisms leading to increased sensitivity to photo-damage (Roth & Deheyn, 2013).

Regeneration, or the renewal or repair of cells, tissues and organs, is widely distributed yet highly variable among metazoans (Alvarado & Tsonis, 2006; Sanchez-Alvarado, 2000; Somorjai et al., 2012). Colonial invertebrates including corals, have the capacity to regenerate entire organisms from tissue fragments, dissociated tissues and even re-aggregated cells (Bosch, 2007; Gierer et al., 1972; Morgan, 1898). Regeneration is classified into two general categories according to the following criteria: (1) regeneration in the absence of cell proliferation and (2) regeneration mediated by cell proliferation (Sanchez-Alvarado, 2000). The first, coined *morphallaxis* is most common in invertebrates, involving the renewal of missing body parts through the remodelling of pre-existing cells. Although cell differentiation is active, the result is a complete individual derived entirely from the reorganisation or exchange of cells from the original organism (Gierer et al., 1972; Chera et al., 2009). *Hydra* regeneration demonstrates the capacity to regenerate without the creation of new material (Bosch, 2007). When individual (non-colonial) *Hydra* polyps are severely damaged, the remaining structures are remodelled to regenerate whole body parts including the mouth and tentacles (Agata, Saito & Nakajima, 2007; Böttger & Alexandrova, 2007; Passamaneck & Martindale, 2012). In corals, regeneration following injury has been documented to occur via both *morphallaxis*, the re-arranging of pre-existing cells in the polyps underlying fine tissue; or *epimorphosis*, involving a cascade of differentiation and proliferation (Harrison, 1972; Fisher et al., 2007; Meesters, Pauchli & Bak, 1997). However injury recovery and tissue repair in corals is impeded by thermal stress (Baker, Glynn & Riegl, 2008; Meesters & Bak, 1993; Meesters & Bak, 1994; Baird & Marshall, 2002). Here we monitored tissue repair, GFP fluorescence, and gene expression patterns in corals for 12 days following injury (removal of apical tips) whilst held under both ambient (26 °C) and sub-bleaching thermal stress (32 °C). In doing so, the study aimed to investigate the impact of sustained exposure to sub-lethal (pre-beaching) temperatures on coral capacity to recover from injury.

## Materials and Methods

**Figure 1:**
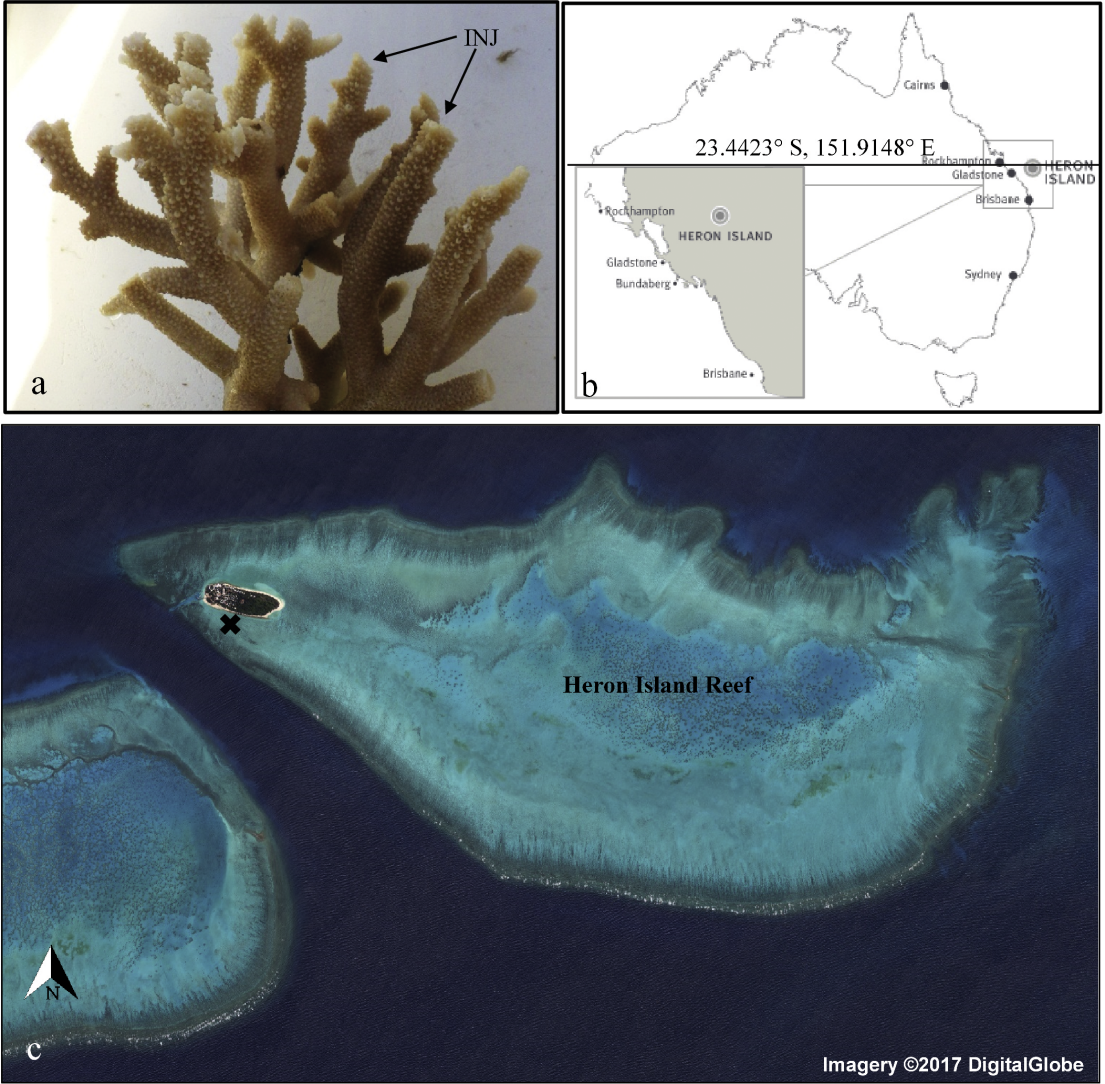
Field site of sample collection of *A. aspera*. Experimental species *A. aspera* following collection and removal of apical tips on 50% of colony branches (A), location of study site, Heron Island (B) and map of Heron Island reef from which experimental coral colonies were collected (X) (C) Map Data: Google Maps, DigitalGlobe.

Large coral colony fragments (*n* = 20) of approximately 20 cm in diameter were randomly sampled (under Great Barrier Reef Marine Park Authority permit number G13/36402.1) from 4 distinct patches of *Acropora aspera* (tan morph) on the Heron Island reef flat (23°26′36.2″S 151°54′43.1″E) at low tide during March 2014 (Fig. 1), no genotyping was conducted. Sampling was conducted at a depth of 0.3 m, temperature range of 26–32 °C, and a midday light intensity between 300 to 2,200 µmol quanta m^−2^ s^−1^. Colony fragments were randomly assigned to two 1,000 litre flow-through seawater mesocosms (<100 m from the site of collection), 10 colony fragments were assigned to each system, left to acclimate for 7 days with flow-through seawater sourced directly from the adjacent reef flat at a high turnover rate, and monitored for recovery. Water in-flow and circulation was identical in the mesocosms and monitored throughout the day for the duration of the experimental period. All corals recovered from fragmentation, corals were observed to be feeding and damage points from collection fully healed during the acclimation, resulting in 20 healthy coral colonies to be used for experimental purposes. To mimic expected reef flat light and temperature regimes, both mesocosms were positioned in full sunlight with no direct shading from adjacent structures. Coral colony fragments were also positioned 20 cm apart from each other, and internal pumps were used to provide sufficient flow within and around the coral colony fragments.

Two days prior to the end of acclimation, the branches of half the colony colonies in each mesocosm (*n* = 5 coral colony fragment colonies) were injured by having apical tips removed (∼3 cm of the apical region) and the remaining five colonies had their tips left intact (Fig. 1). The corals where maintained in ambient conditions for two days to allow wound healing to initiate and corals were monitored for health throughout this period. This design was applied to provide a control baseline comparison of molecular regulation of heat-shock responses between the two mescosms (n,10 colonies) without the application of sub-lethal thermal stress, and allow for within treatment controls to compare coal recovery under the two thermal regimes. Following the acclimation period one mesocosm was maintained at ambient inflow seawater conditions of 26 °C while the other mesocosm had incoming seawater increased to 32 °C (sub-bleaching) during daylight hours over a 12-day period.

Coral colonies showed no visible signs of paling or mucous sloughing when exposed to daily peak temperatures of sub-bleaching 32 °C (2 degrees below the bleaching threshold of 34 °C for *A. aspera* (Ainsworth et al., 2016)) with changes in tissue pigmentation monitored twice daily and compared to coral health check colour cards. To replicate natural reef temperature conditions, aquarium heaters in the temperature treatment mesocosm (32 °C) were switched off at 1,900 h and switched on from 0,700 h each day for a duration of 12 h. Seawater temperature was recorded using Odyssey temperature loggers (Christchurch, New Zealand) every two minutes. Light levels in both systems were monitored every 10 min with Odyssey Photosynthetic Active Radiation (PAR) recorders (Dataflow Systems Limited, Christchurch, New Zealand), ranging between 700–1,500 µmol quanta m^−2^ s^−1^.

On days 2, 4, 6, 8, 10 and 12 following removal of the apical tips, 4 branches from each coral colony fragment were randomly sampled at mid-day (1200 h) from each of the two treatments. Coral branches (*n* = 2 of 4) were fixed with 4% paraformaldehyde (PFA) and 3 × Phosphate Buffer Saline (PBS) overnight, then transferred and stored in 3 × PBS at 4 °C. The remaining coral branches (*n* = 2 of 4) were also immediately snap-frozen in liquid nitrogen (LN_2_) and stored at −80 °C. Coral branches snap frozen in liquid nitrogen were crushed using a hydraulic press and homogenised to a fine powder in a mortar chilled with liquid nitrogen under RNase free conditions. Samples were then stored at −80 °C for mRNA purification using the Dynabeads^®^ mRNA DIRECT™ Kit (Ambion, Foster City, CA, USA) and DNase/cDNA synthesis. Messenger RNA was isolated from approximately 150 mg of crushed sample from injured (*n* = 120) and non-injured (*n* = 120) coral branches using the Dynabeads^®^ mRNA DIRECT™ Kit (Ambion, Foster City, CA, USA) (as per manufactures instructions). Isolated mRNA was quantified spectrophotometrically using a NanoDrop-1000 (NanoDrop Technologies, Wilmington, DE, USA). Genomic DNA contamination was removed from 0.1 µg of mRNA via a DNase digest treatment using the RQ1 RNase-Free DNase (Promega, Madison, WI, USA). Complementary DNA (cDNA) was synthesised from DNase-treated mRNA for use in quantitative real-time-PCR (qRT-PCR). Approximately 100 ng of mRNA was reverse transcribed using the SuperScript™ III First-Strand Synthesis (SSIII) SuperMix for qRT-PCR (Invitrogen), according to manufacturer’s instructions, with oligo (dT) primers.

Quantitative real-time PCR was performed using a Rotor-Gene™ 6000 (Corbett Life Sciences, New South Wales, Australia) robot and a CAS-1200 robotic liquid handling system (Corbet Robotics, Australia). A final volume of 15 µl was analysed in the PCR, containing 7.5 µl of GoTaq^®^ qPCR Master Mix (Promega, Madison, WI, USA), 3.5 µl of gene specific primers and 4 µl of dilute cDNA template. Quantitative PCR was performed under the following conditions: a 1 cycle hold-start activation at 95 °C for 2 min, followed by 40 cycles of 15 s at 95 °C and 60 s at 60 °C, with a melt stage between 55 °C and 95 °C with a temperature increase of 1 °C every 5 s. General stress response genes were quantified to confirm corals’ were undergoing thermal stress responses (heat shock protein 70 (HSP70), heat shock 90 (HSP90) and Catalase). In addition to the genes of interest, Adohcyase (Ado) and Ctg1913 were used as coral housekeeping genes (HKGs) (Table 1). Each Rotor-Disc™ 100 (Qiagen, Hilden, Germany) included three technical replicates for 10 biological samples with two template/housekeeping genes. Technical replicates were automatically averaged when CT values were within 1 CT. In the few cases were this was not the case amplification curves of the replicates were examined manually, and replicates removed where amplification did not occur. Samples were repeated if filtered technical replicates were not with one CT value. Non-template controls for all runs were performed in triplicate.

**Table 1:** Oligonucleotide sequences of target and housekeeping genes. Oligonucleotide primer sequences of target and housekeeping genes for RT-qPCR of *A. aspera*.

Gene name	Oligonucleotide primer sequence (5′–3′)	Cited source
*Target genes*		
HSP70	F_1_:AGGAGACCGCTGAGGCATACTTG *R*_1_:CTTGGTGGCCTGACGCTGAGAATC	Ogawa et al. (2013)
HSP90	F_1_:ATTCCGAGGATCTGCCACTGA R_1_:TCTCTGCGATCTCTGCGAACAT	Ogawa et al. (2013)
Catalase	F_1_:GCAAAGTAGTTGGACGCGTTAC R_1_:GGAATCCTTTCGACCTCACTAAG	Seneca et al. (2010) and Ainsworth et al. (2016)
*Housekeeping genes*		
Ctg1913	F_1_:GATTTAACCACCGGCAGTGT R_1_:ATGGTAGGGAGGAGGCTGTT	Ogawa et al. (2013) and Ainsworth et al. (2016)
Ado	F_1_:AAGAAGACAAACATCAAGCCTCA R_1_:CACATCCAAGGTTCACAAGACG	Ogawa et al. (2013)

Template cDNA dilution series were prepared to optimize quantification accuracy. Serial dilutions between 1/5 and 1/80 were performed on a composite of cDNA randomly selected from a variety of thermal regimes over the course of the experiment. These dilutions were used to construct standard curves for the three target genes, HSP70, HSP90 and Catalase; and two housekeeping genes, Ado and Ctg1913. For analysis, cDNA was diluted 1/10 prior to use as a template in qRT-PCR analysis. Standard curves for each gene were imported into qBASE plus 2.5 software (Biogazelle; http://www.biogazelle.com/products/qbasePLUS) for CT value normalisation by calculating the geometric mean of the housekeeping genes. Relative expression analysis were then performed using qBASE. Statistics software package SPSS (SPSS Statistics v 22.0, IBM, Armonk, North Castle, NY, USA) was used for all statistical analyses. Significant differences in the relative expression of heat shock protein, HSP70 and HSP90; and antioxidant genes, Catalase, were determined using a generalised linear model. ‘Injury’ and ‘temperature’ were both considered as single factors with two levels. Factor ‘day’ was accounted as a repeated measure. For significantly different data the sequential Bonferroni *post-hoc* least significant difference (LSD) test was performed to adjust for type I error.

PFA fixed coral tips were viewed and photographed under an Olympus stereoscopic microscope (Olympus SZX16^®^, SDF PLAPO; Olympus Corporation of America, Center Valley, PA, USA). Photographs of the apical and lateral polyps were taken using a Fujifilm MX-2900ZOOM digital camera, under both visible light and GFP epifluorescence (Excitation wavelength = 460–490 nm; Emission wavelength = 510 nm). GFP fluorescence at the injury site was quantified using image analysis software ImageJ by calculating Corrected Total Cell Fluorescence (CTCF = Integrated Density − (Area of selected cells × Mean Fluorescence of background readings)) (McCloy et al., 2014; Burgess et al., 2010). Following visible and epifluorescence microscopy, the PFA fixed coral tips were then bleached using a 1:1 ratio of 4% hypochlorite bleach (Brighton Professional) to tap water to remove all biological material, followed by three washes in tap water to remove any residual calcified crystals. The calcified tips were then placed onto aluminium mounts using the plastic conductive carbon cement Leit-C-Plast (ProSciTech, Australia™), and coated in six layers of gold. Samples were viewed and photographed using scanning electron microscopy (SEM) (JOEL, Tokyo, Japan, model JSM5410LV) at 15× and 5.0 kv magnification to compare topographical differences in apical tip growth under ambient verses temperature-treated (sub-bleaching) conditions over the 12-day experimental period. Linear extension of the regenerating apical tips was measured from a fixed landmark (centre point on the cut site at the base of the apical tip to the top of the extending apical tip) using ImageJ, with coral branches positioned in the same direction in each image. Repeated measures ANOVAs were performed using SPSS (SPSS Statistics v 22.0, IBM, USA) to compare (1) differences in linear extension (mm day^−1^) and (2) differences in GFP fluorescence intensity (CTCF) between ambient and temperature stressed treatments.

## Results

### Visible and GFP epi-fluorescent response to injury and thermal stress

**Figure 2:**
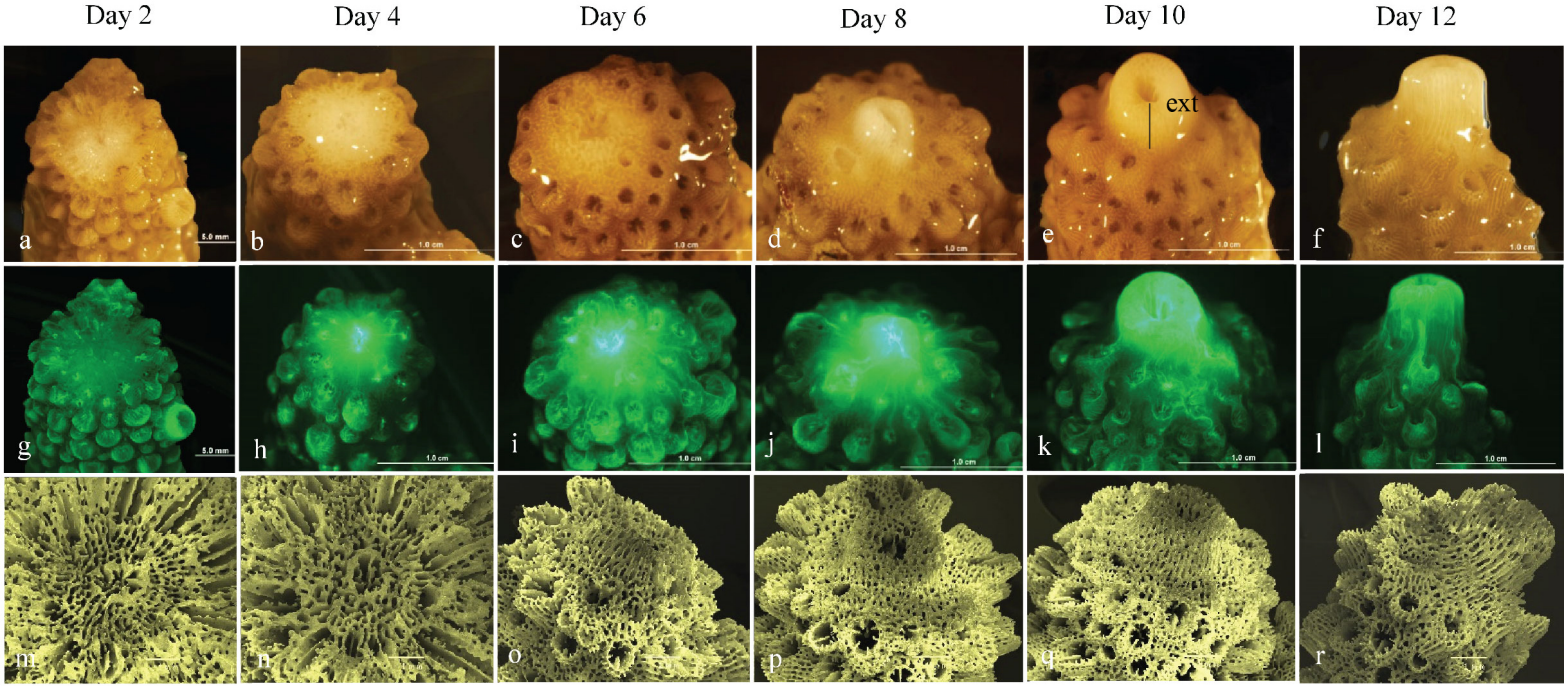
Microscopic examination of apical tip regeneration following exposure to ambient SST conditions. Time series photograph (A–F), epi-floruescence photographs (G–L) and scanning electron micrograph of skeletal structure (M–R) of *A. aspera* injured corals held under ambient SST conditions (26°C). Measure of apical tip extension denoted by (ext).

**Figure 3:**
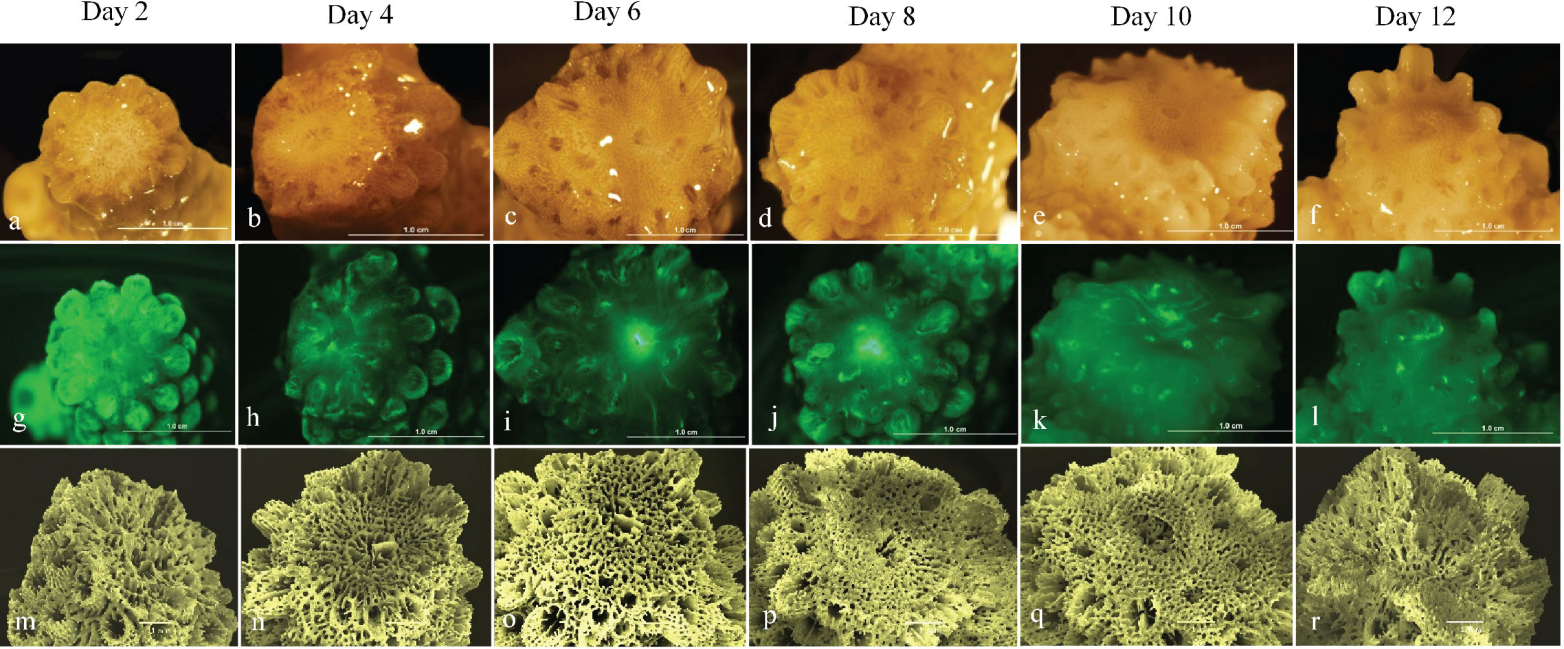
Microscopic examination of apical tip regeneration following exposure to elevated SST conditions. Time series photograph (A–F), epi-floruescence photographs (G–L) and scanning electron micrograph of skeletal structure (M–R) of *A. aspera* injured corals held under elevated SST conditions (32°C).

**Figure 4:**
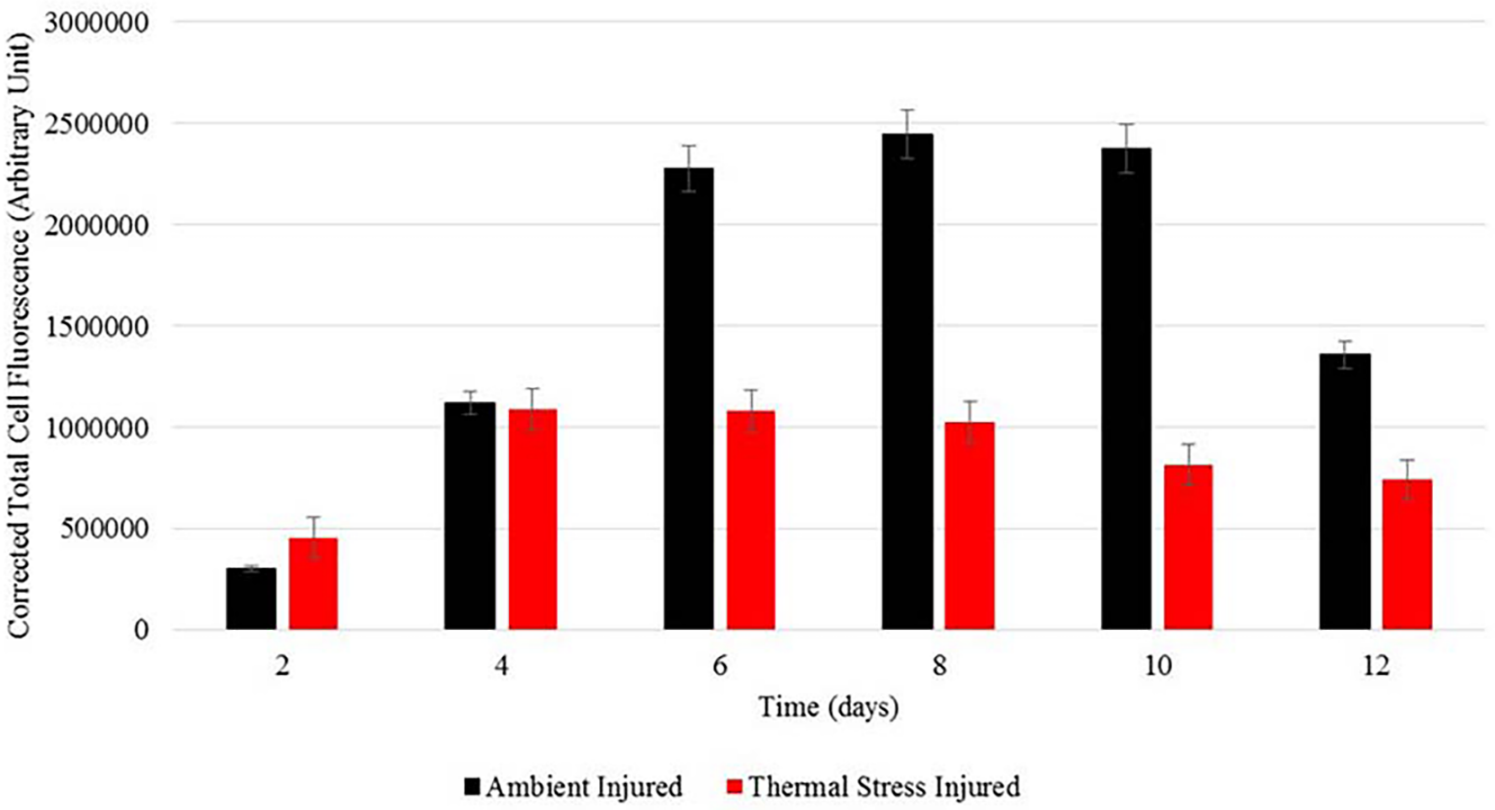
Corrected Total Cell Fluorescence. Average Corrected Total Cell Fluorescence (CTCF) of injury site (i.e., site of apical tip removal) in *A. aspera* held under ambient (26°C) and elevated (32°C) SST conditions, Mean = ±1 s.e.

The regenerative and wound healing response of experimentally injured *A. aspera* to the synergistic impacts of thermal stress was documented by a time-series of micrographs of the apical tips of colonies held under temperature stressed and ambient conditions. Examination of colonies with their apical tips removed, revealed significant apical regeneration/healing under ambient summertime seawater conditions (26 °C) (Figs. 2A–2F). More than 50% of regrowth of the apical tip had occurred by day 8 and complete regrowth of the apical tip was evident by experimental day 12. Epifluorescence examination of apical tips of corals under ambient conditions further revealed that GFP fluorescence was evident at the wound site where the apical tip had been removed and within the reforming apical tip (Figs. 2G–2L). GFP fluorescence measured via the CTCF method (McCloy et al., 2014; Burgess et al., 2010) was significantly different for corals exposed to ambient and temperature stressed conditions (Wilks’ Lambda = 0.42, *F*_1,5_ = 6.8, *p* < 0.05). GFP fluorescence increased during apical tip regrowth between day 2 (∼300000 CTCF) and 10 (∼2500000 CTCF) under ambient seawater conditions and was observed within the newly forming coral apical tip throughout the experimental period. During exposure to sub-bleaching seawater temperature conditions, increased GFP fluorescence was only evident at the central polyp growth channel on the wound site at day 4 (∼1100000 CTCF), 6 (∼1100000 CTCF) and 8 (∼1000000 CTCF) (Figs. 3H–3L) (Fig. 4).

### Calcification response to injury and thermal stress

**Figure 5:**
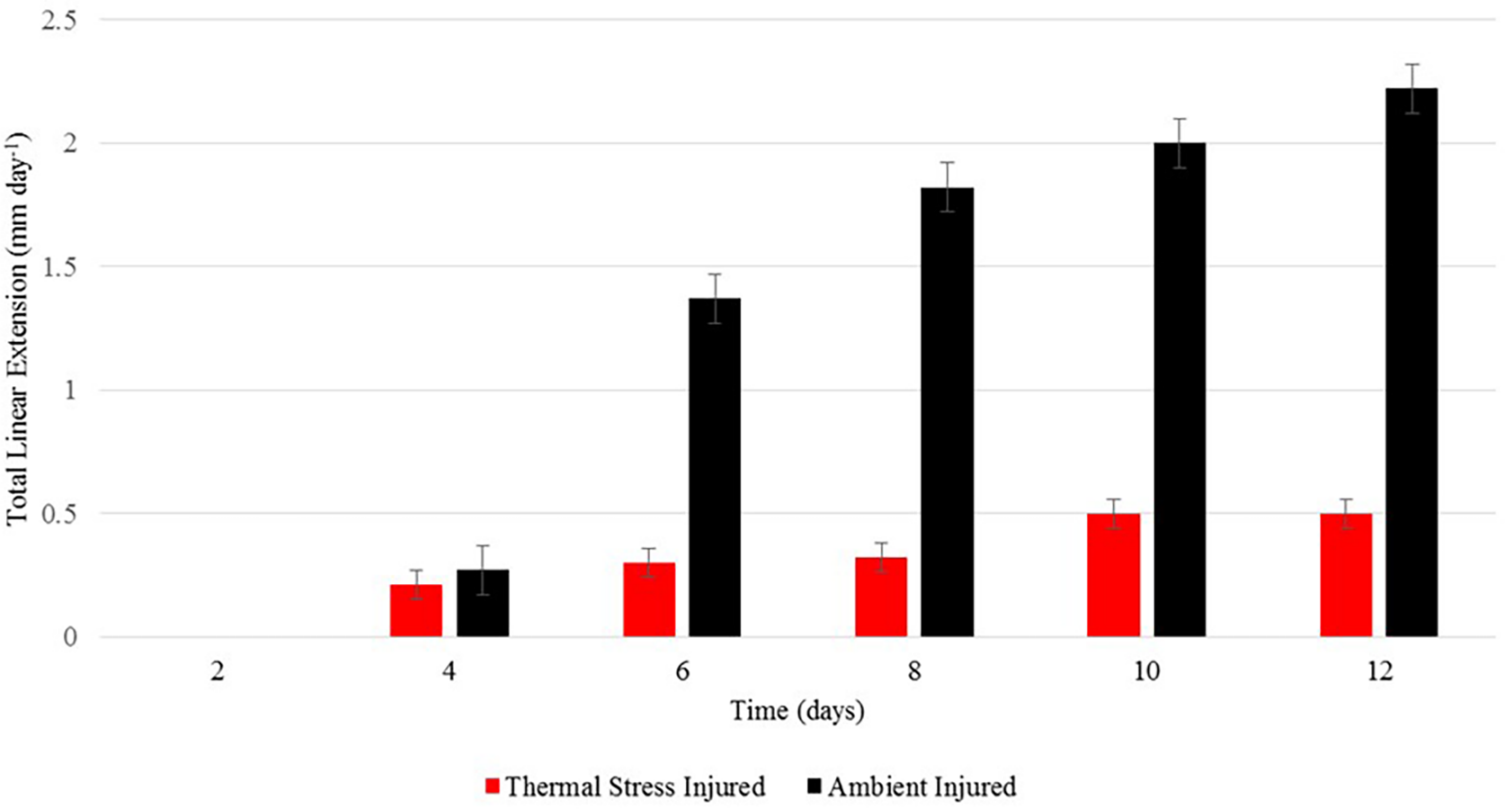
Linear extension rates. Average total linear extension (mm per day^−1^) of apical tips in *A. aspera* held under ambient and elevated SST conditions, Mean = ±1 s.e.

Scanning electron microscopy was used to examine the effect of sub-bleaching thermal stress on skeletal regeneration of the coral apical tips. Exposure to sub-bleaching conditions (32 °C) had a visible effect on patterns of skeletal regeneration across the 12-day experimental period. Un-injured corals sampled from days 2 and 12 were used as base-line comparisons of intact corallite structure. Skeletal linear extension (mm per day^−1^) of the coral apical tip was significantly different between temperature stressed and ambient seawater conditions (Wilks’ Lambda = 0.33, *F*_1,5_ = 9.8, *p* < 0.05) (Fig. 5). During exposure to ambient seawater conditions, ∼1.44 mm extension of the calcium carbonate skeletal was evident by day 6 with complete extension of ∼2.2 mm evident by day 12. Under exposure to sub-bleaching temperature stress an emergent apical tip was only evident in some samples by day 10 with only ∼0.5 mm extension of the apical tip evident.

### Gene expression analyses

**Figure 6:**
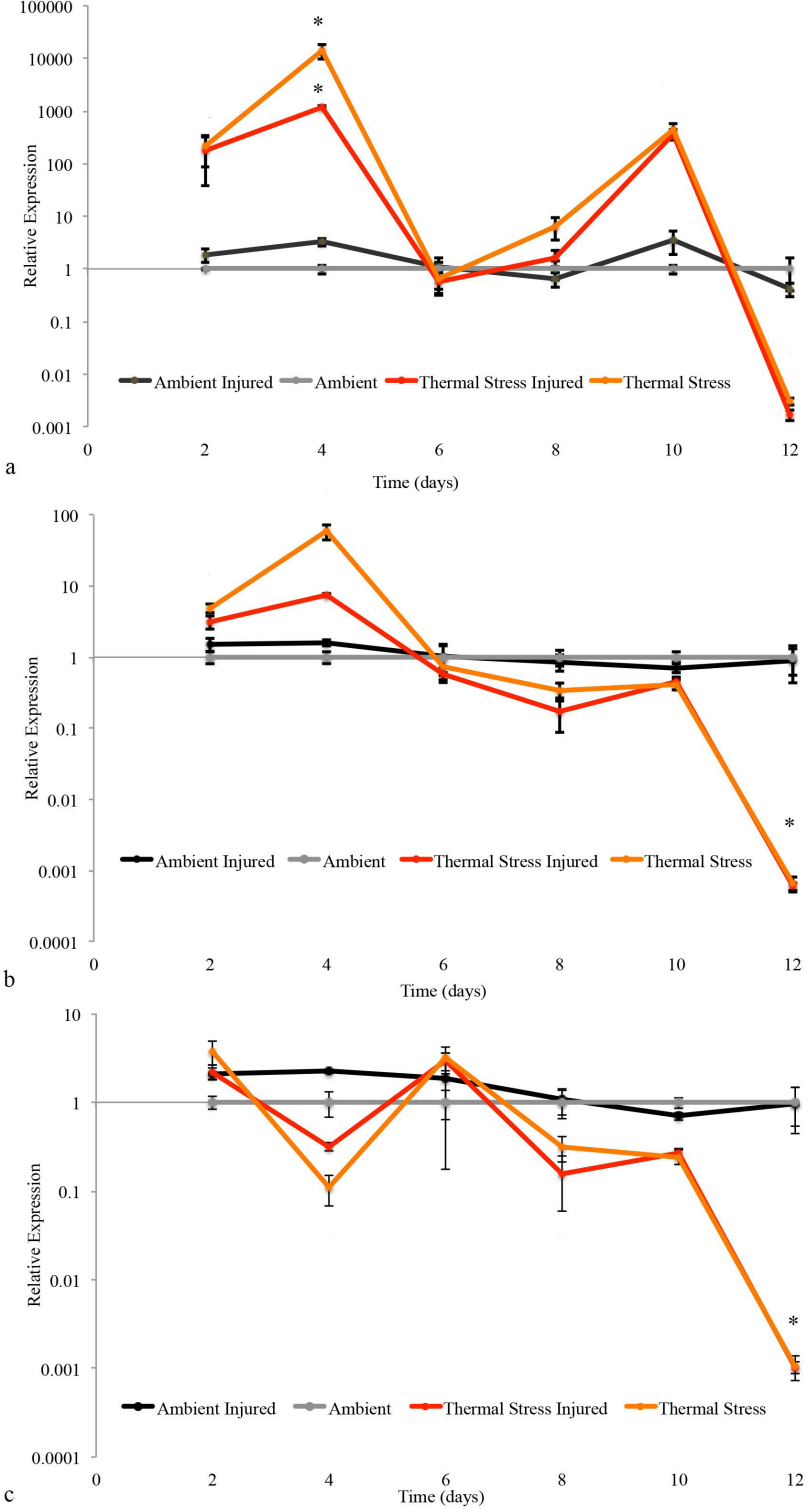
Relative gene expression of target genes. Relative expression of injured and un-injured *A. aspera* Catalase (A), HSP 90 (B), and HSP 70 (C) in corals held under ambient (26 °C) and elevated (32 °C) SST conditions, Mean = ±1 s.e.

Heat shock protein and catalase regulation were examined in the coral host throughout the experimental period. Both temperature and injury had a significant effect on catalase expression resulting in a down-regulation at day 4 (14,069.56 fold, *p* < 0.001). There was also a significant interaction of temperature and injury at day 4 on catalase expression. Under ambient (control) conditions on this day, there was no significant difference in expression between injured and non-injured corals. Instead, at elevated temperatures, there was a significant difference in expression between injured and non-injured corals (1000.1 fold, *p* < 0.001) (Fig. 6A). The response of both HSP70 and HSP90 was determined for the coral *A. aspera* over the course of the 12 day experimental period. In the coral host, temperature had a significant effect on HSP70 and HSP90 expression, resulting in a down-regulation at day 12 (979 fold decrease, *p* < 0.001; 1,676 fold decrease, *p* < 0.001). However, there was no significant interaction between temperature and injury on both HSP90 (Fig. 6B) and HSP70 expression (Fig. 6C).

## Discussion

In scleractinian corals injury results in the removal of tissue and/or skeletal from the colony. Injuries are generally categorised based on the amount of tissue lost, including partial or total tissue injuries, and superficial (e.g., scrapings/gross lesions) or extensive (e.g., bump/branch removal) for injuries resulting in functional and morphologic changes in tissue and skeleton (Stafford-Smith, 1993; Hall, 1997). Regeneration/wound healing following extensive injury has been documented to occur in >74 days post-injury, and between 24 h and 80 days following partial injuries (Hall, 1997; Bak, 1983; Meesters & Bak, 1994; DeFilippo et al., 2016). Here we find the recovery and regeneration of *A. aspera* to injury (i.e., apical tip removal) was significantly impeded following exposure to sub-bleaching thermal stress conditions (32 °C) compared to summertime ambient seawater conditions (26 °C). Injured corals showed less than 50% apical tip regrowth occurring within 12 days post-injury. Previous studies have shown that corals compromised by thermal stress have reduced growth rates (Leder, Szmant & Swart, 1991) and consequently lower tissue regeneration capacities (Meesters & Bak, 1993). This is supported by Denis et al. and colleagues (2013) who reported a significant reduction in the regeneration/recovery of superficial lesions on *A. muricata* nubbins at sites exposed to high fluctuating seawater temperatures (∼192 days post-injury) as opposed to sites characterised by stable seawater temperatures (∼81 days post-injury). Damaged juvenile colonies of *Porites spp*. were also found to show impeded regeneration at sub-lethal 29.6 °C compared to colonies exposed to 26.7 °C (Edmunds & Lenihan, 2010). Similarly, comparison of wound healing in naturally non-symbiotic and symbiotic *Astrangia poculata* found that the presence of *Symbiodinium* increased wound healing ability (DeFilippo et al., 2016; Burmester et al., 2017). In view of this, our observations suggest that exposure to sub-bleaching thermal stress (32 °C) also impairs the healing capacity of *A. aspera* due to a reduced availability of energetic resources and that the cellular regeneration and repair mechanisms may be compromised by the physical environment and the direct effects of temperature on coral metabolism and growth (as described by Kramarsky-Winter & Loya, 2000; Jokiel, 2004; Edmunds & Gates, 2008). Studies with additional mesocosms and temperature conditions could further characterise the dynamics of this synergistic interaction.

### Calcification response to injury and thermal stress

Exposure to sub-bleaching thermal stress (32 °C) had a visible impact on skeletal formation at the coral apical tip, with linear extension only evident in some samples by day 10 compared to full extension being evident by day 12 in corals held in normal, ambient, seawater conditions (26 °C). Calcification and extension rates in corals are significantly greater at the top of colonies (i.e., branch tips) than at the sides (Lough & Barnes, 2000). When subjected to thermal stress, reef-building corals have been shown to exhibit abnormally reduced extension and calcification rates, and are more susceptible to other stressors including bleaching (Goreau & Macfarlane, 1990); Meesters & Bak, 1993; De’ath, Lough & Fabricius, 2009; Cantin et al., 2010). Previous studies have postulated an increase in calcification rates with increasing temperature up to an optimum and then declining significantly as water temperatures approach ∼30 °C, likely the result of increasing levels of thermal stress (Jokiel & Coles, 1977; Marshall & Clode, 2004). Our results, in conjunction with what has been reported in previous literature, indicate that exposure of *A. aspera* to synergistic sub-bleaching thermal stress (32 °C) substantially reduces skeletal extension in the coral apical tips. It is also possible that physiological adjustments (i.e., higher rates of heat shock protein production and/or ROS scavenging) could exert energetic costs high enough to subvert calcification (Dandan et al., 2015), thus explaining the reduced calcification and extension in the *A. aspera* apical tips when subjected to sub-bleaching thermal stress.

### GFP epi-fluorescent response to injury and thermal stress

GFP epi-fluorescence was found to be significantly lower in injured corals under thermally stressed seawater conditions (32 °C), compared to ambient seawater conditions (26 °C). In the current study GFP fluorescence in the apical tips was found to significantly increase between days 4 and 12 following injury under normal ambient seawater conditions (26 °C), but an increase in GFP at the injury site was absent in thermally stressed, injured, corals. Simultaneous exposure to thermal stress and/or solar radiation in shallow tropical waters such as that exposed to *A. aspera*, is known to cause photoinhibition and increase the production of damaging ROS in *Symbiodinium* and coral host tissue, and lead to perturbations of the metabolic and cellular processes in *Symbiodinium* and/or their coral host cells (Smith, Suggett & Baker, 2005; Bou-Abdallah, Chasteen & Lesser, 2006) due to the overwhelming of antioxidant defences. In *A. aspera,* GFP-like homologs are also hypothesised to be photo-protective at normal temperatures less than 32 °C (Dove, 2004). The observed increase in GFP fluorescence may be due to the photo-protection of newly differentiating host tissue from the build-up of harmful ROS elevated in the epithelial and gastrodermal tissue during injury, in addition to the deposition of a newly forming calcium carbonate skeleton. A high concentration of host pigments in this active growth region acts to deflect visible, infra-red light and thermal damage from the coral host tissue surfaces (Bou-Abdallah, Chasteen & Lesser, 2006; Salih et al., 2000; Kaniewska et al., 2012), however this protection does not occur under temperature regimes which result in thermal stress to the coral.

### Antioxidant gene expression

Endogenous antioxidant enzymes including catalase and superoxide dismutases are a corals first line of defence against the harmful effects of excessive ROS production in *Symbiodinium* and host tissues. The sensitivity of these enzymes to temperature makes them particularly useful indicators for the onset of thermal stress and bleaching in reef-building corals (Seneca et al., 2010; Merle et al., 2007). In the current study, exposure of *A. aspera* to sub-bleaching thermal stress (32 °C) was characterised by a significant up-regulation in catalase expression at day 4. In corals, up-regulation of catalase is important in limiting the production of highly cytotoxic hydroxyl radicals (Ross et al., 2010). Our findings in *A. aspera* following thermal stress are consistent with previous studies in various species of adult and juvenile corals, reporting consistent up-regulation within 48 h following immediate exposure to increased seawater temperatures (Voolstra et al., 2009; Pernice et al., 2011).

Catalase expression was also found to show significant up-regulation at day 4 in response to injury. Coral tissue compromised by injury, exhibit an increased abundance of damaging oxygen radicals, due to increased activity of the melanin-synthesis pathway (Palmer, Modi & Mydlarz, 2009). This may therefore have resulted in the induction of catalase as a scavenger of ROS at the site of injury, resulting in a significant up-regulation. Further evidence may be drawn from higher metazoan species and plants. Following traumatic brain injury in rats, catalase showed significant upregulation 3 days post-injury in comparison to non-traumatised controls. This was followed by a return to normal expression by day 7 (Goss et al., 1997). Given the findings in *A. aspera*, peak expression was found at day 4 followed by a drop in expression level at day 6. Findings by Guan, Zhao & Scandalios (2000) have also reported up-regulation of three catalase homolog genes in response to wounding in immature embryos and leaves of maize, a response to increased levels of endogenous H_2_O_2_ in wounded tissue and leaves. Importantly, a significant temperature stress/injury interaction effect on catalase up-regulation was found only under thermally stressed seawater temperatures. During the coral stress response, the effects from temperature stress, injury and/or infection contribute to elevated ROS in the coral host tissue (Palmer, Modi & Mydlarz, 2009). Hence, it can be postulated that the combination of sub-bleaching temperature and injury in *A. aspera* were responsible for an up-regulation in catalase activity in response to overcome the overproduction of damaging ROS at the injured apical tips.

### HSP gene responses

The role of HSPs as molecular chaperones are to aid in proper protein folding and prevent aggregation at elevated temperature and other stressors (Sørensen, Dahlgaard & Loeschcke, 2001), and are generally normally up-regulated by organisms under heat stress (Feder & Hofmann, 1999). It is therefore unusual that no significant up-regulation of HSP70 or HSP90, following exposure to sub-bleaching thermal stress in *A. aspera,* was observed during this study. Despite this, a significant down-regulation was reported only at day 12. Several studies examining thermal stress in a diversity of coral species have failed to detect an up-regulation in either genes. Desalvo et al. (2008) reported no up-regulation in HSP70 in thermally stressed *M. faveolata*, while Mayfield et al. (2011) found no differential expression of HSP70 in heat stressed *Seriatopera hystrix*. Lack of differential expression in the HSPs may have resulted from not sampling earlier enough to capture expressional changes following the induction of thermal stress (Bellantuono et al., 2012). Studies on the effect of heat stress on *A .millepora* larvae supports this interpretation, with the transcriptional induction of HSP70 and HSP90 detected after only 3 h (Rodriguez-Lanetty, Harii & Hoegh-Guldberg, 2009). However, it is important to acknowledge that a rapid induction of HSPs following heat stress has been reported in several coral species including *A. grandis*, *M. faveolata* and *Goniopora djiboutiensis*.

## Conclusion

Understanding corals’ capacity for recovery, the drivers of a failure to recover from injury, and the underlying mechanisms governing coral regeneration/injury repair, are particularly important given the projected change to coral reefs under future climate change. Mass bleaching events, attributed to increasing SSTs and solar irradiance (e.g., UV and IR), have driven a worldwide decline of 30% in coral cover (Harrison, Dubinsky & Stambler, 2011; Hill & Wilkinson, 2004; Hoegh-Guldberg et al., 2004). Despite this, the underlying molecular and cellular mechanisms controlling regenerative/repair processes under thermal stress have not been thoroughly investigated. In addition, recent work has demonstrated that the coral immune system assists in protecting regenerating tissue from microbial competition (Van de Water et al., 2015a; Van de Water et al., 2015b), which is particularly important considering how microbial populations may shift as climate changes (Ainsworth & Gates, 2016). This study was the first to demonstrate a synergistic impact of temperature and injury to coral recovery, regrowth and skeletal regeneration in Acroporid corals. In doing so, this study also highlights the need for further research into both synergistic and sub-bleaching stressors to further determine the impact of climate change on coral reef ecosystems.

##  Supplemental Information

10.7717/peerj.3719/supp-1 Supplemental Information 1 Raw dataset Raw gene expression statistics for Catalase, HSP90 and HSP70.

10.7717/peerj.3719/supp-2 Supplemental Information 2 CTCF raw dataset statistics

10.7717/peerj.3719/supp-3 Supplemental Information 3 Linear extension rate dataset statistics

10.7717/peerj.3719/supp-4 Supplemental Information 4 Supplementary microscopy images Visible, GFP and SEM micrograph supplementary images.

## Additional Information and Declarations

### Field Study Permissions

The following information was supplied relating to field study approvals (i.e., approving body and any reference numbers): The Great Barrier Reef Marine Park Authority approved this study (permit number G13/36402.1).

### Data Availability

The following information was supplied regarding data availability: The raw data is supplied as Supplemental Files.
