# Supplementary material for: Exposure to elevated sea-surface temperatures below the bleaching threshold impairs coral recovery and regeneration following injury
Source: PeerJ. 2017 Aug 18;5:e3719. doi: 10.7717/peerj.3719 (PMC5564385; doi:10.7717/peerj.3719)
Supplement: Supplemental Information 3 [file peerj-05-3719-s003.docx]

**Statistical Analyses for linear extension**

| **Multivariate Tests** | | | | | | | | |
| --- | --- | --- | --- | --- | --- | --- | --- | --- |
|  | Value | F | Hypothesis df | Error df | Sig. | Partial Eta Squared | Noncent. Parameter | Observed Power^b^ |
| Pillai's trace | .663 | 9.823^a^ | 1.000 | 5.000 | .026 | .663 | 9.823 | .339 |
| Wilks' lambda | .337 | 9.823^a^ | 1.000 | 5.000 | .026 | .663 | 9.823 | .339 |
| Hotelling's trace | 1.965 | 9.823^a^ | 1.000 | 5.000 | .026 | .663 | 9.823 | .339 |
| Roy's largest root | 1.965 | 9.823^a^ | 1.000 | 5.000 | .026 | .663 | 9.823 | .339 |
| Each F tests the multivariate effect of Time. These tests are based on the linearly independent pairwise comparisons among the estimated marginal means. | | | | | | | | |
| a. Exact statistic | | | | | | | | |
| b. Computed using alpha = .01 | | | | | | | | |

There was a significant difference in linear extension between ambient and temperature injured corals over the 12-day experimental period.

**(F_1_,_5_, p < 0.05, n^2^ = 0.66)**

| **Multivariate Tests^a^** | | | | | | | |
| --- | --- | --- | --- | --- | --- | --- | --- |
| Effect | | Value | F | Hypothesis df | Error df | Sig. | Partial Eta Squared |
| Treatment | Pillai's Trace | .579 | 6.883^b^ | 1.000 | 5.000 | .047 | .579 |
|  | Wilks' Lambda | .421 | 6.883^b^ | 1.000 | 5.000 | .047 | .579 |
|  | Hotelling's Trace | 1.377 | 6.883^b^ | 1.000 | 5.000 | .047 | .579 |
|  | Roy's Largest Root | 1.377 | 6.883^b^ | 1.000 | 5.000 | .047 | .579 |
| a. Design: Intercept  Within Subjects Design: Treatment | | | | | | | |
| b. Exact statistic | | | | | | | |
