# Supplementary material for: Exposure to elevated sea-surface temperatures below the bleaching threshold impairs coral recovery and regeneration following injury
Source: PeerJ. 2017 Aug 18;5:e3719. doi: 10.7717/peerj.3719 (PMC5564385; doi:10.7717/peerj.3719)
Supplement: Supplemental Information 4 — Visible, GFP and SEM micrograph supplementary images. [file peerj-05-3719-s004.pdf]

STEREOSCOPIC MICROSCOPY SUPPLEMENTARY MICROGRAPHS

<u>Day 2 Controls:</u>

Cut **Uncu**

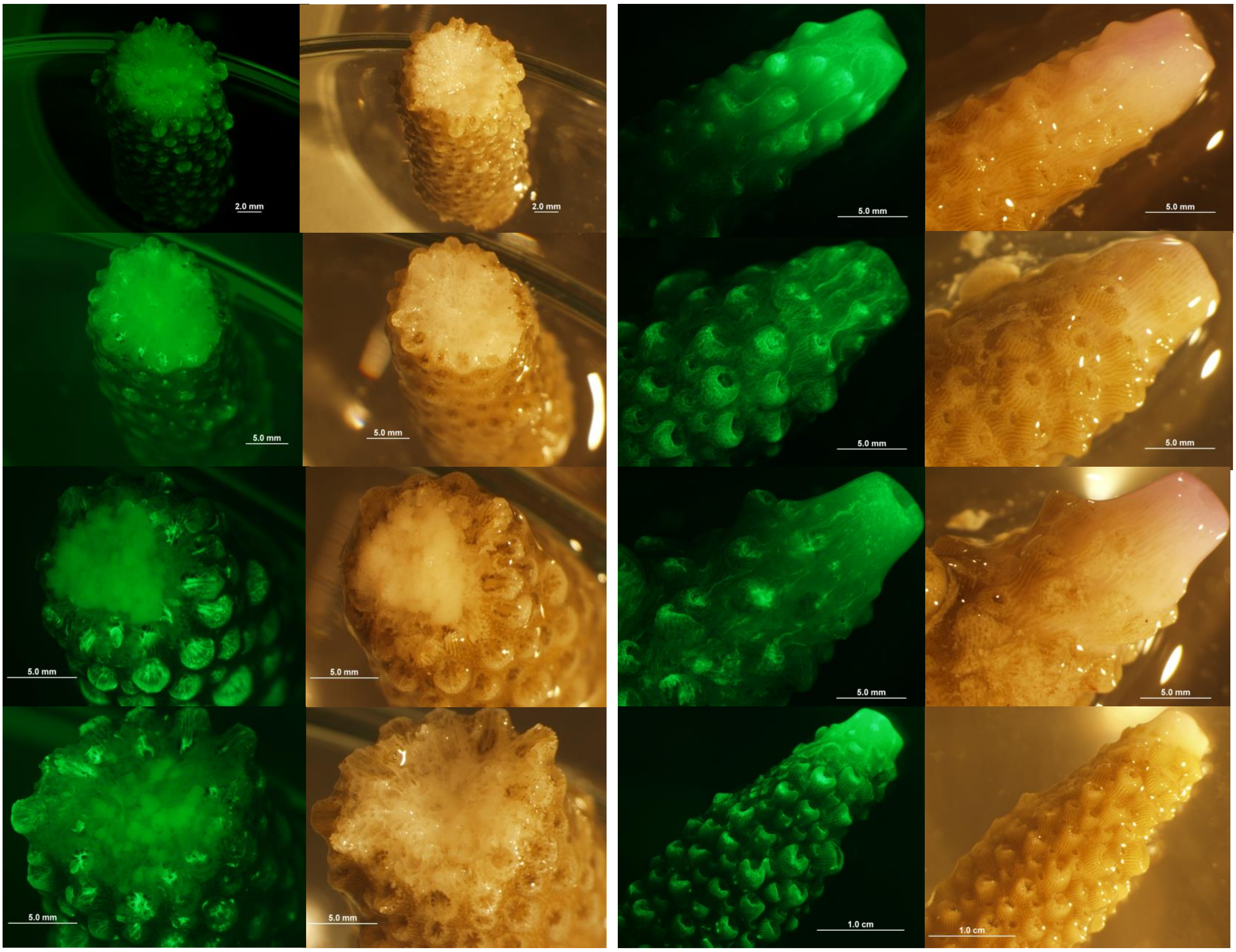

Figure 1. Stereoscopic microcopy images of cut (left) and non-cut (right) apical tips held under controlled seawater conditions (~ 25ºC), day 2.

Day 2 Treatments

Cut **Uncut**

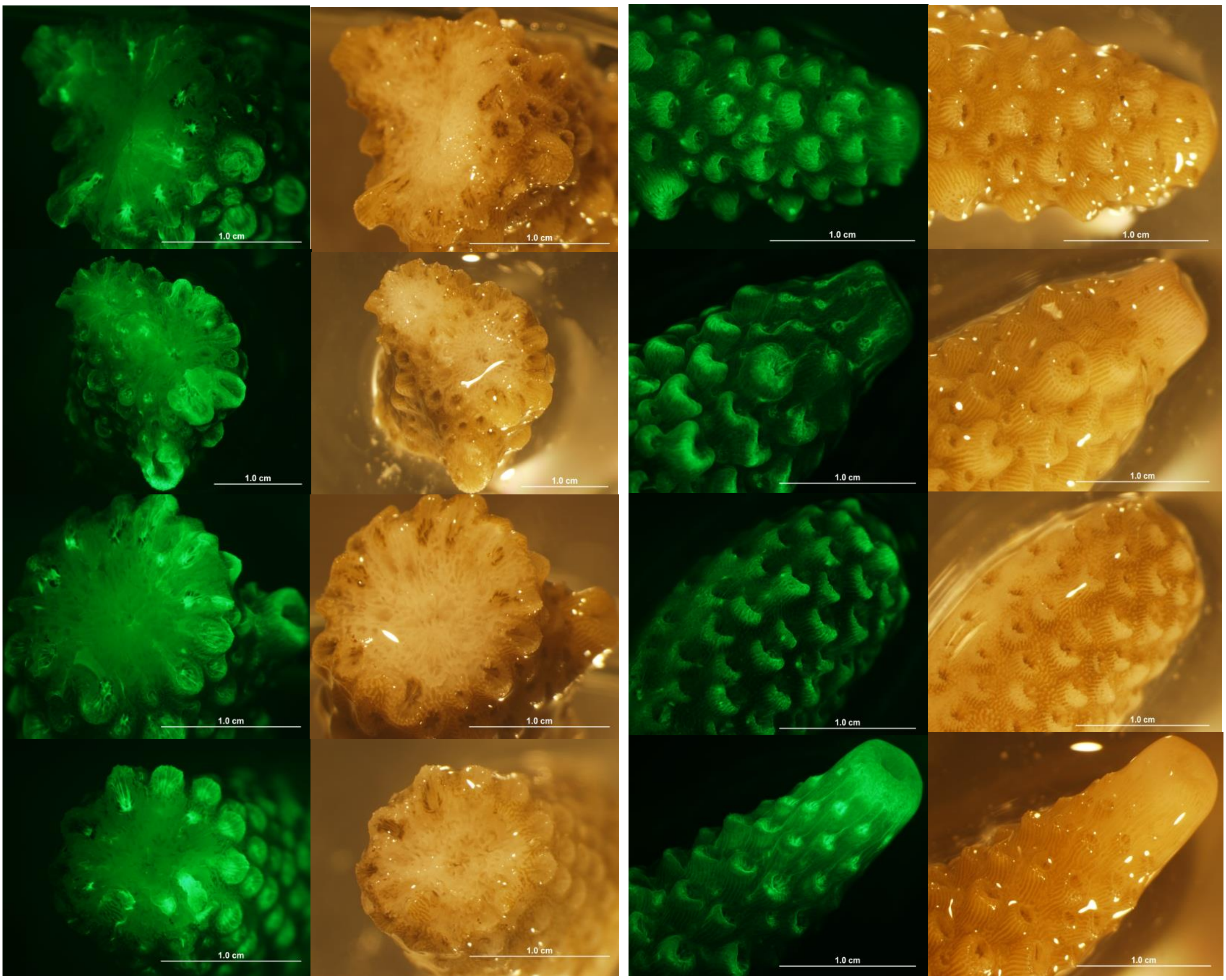

Figure 2. Stereoscopic microcopy images of cut (left) and non-cut (right) apical tips held under treated seawater conditions (~ 32°C), day 2.

Day 4 Controls:

Cut **Uncut**

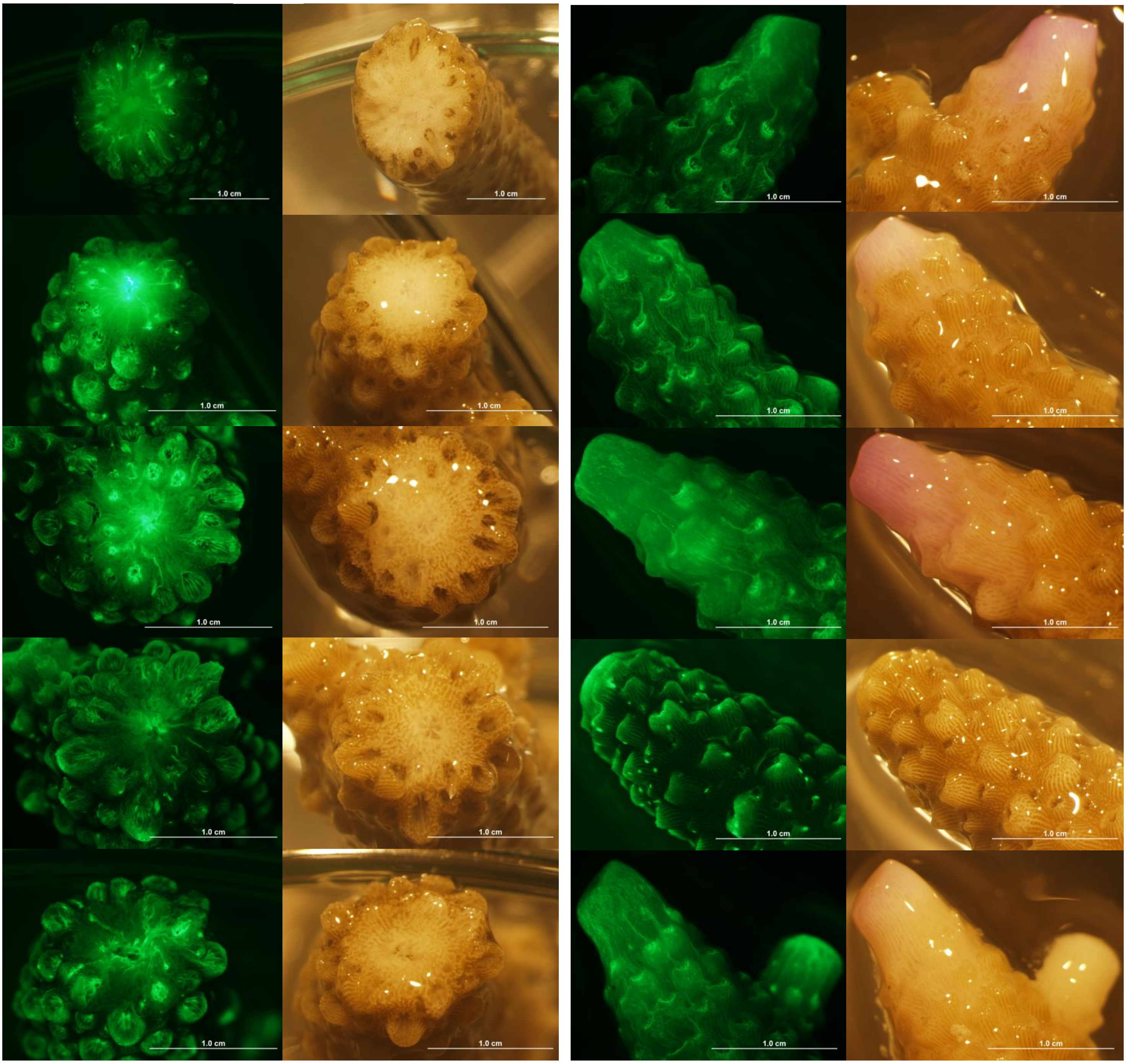

Figure 3. Stereoscopic microcopy images of cut (left) and non-cut (right) apical tips held under control seawater conditions (~ 25ºC), day 4.

Day 4 Treatments:

Cut **Uncut**

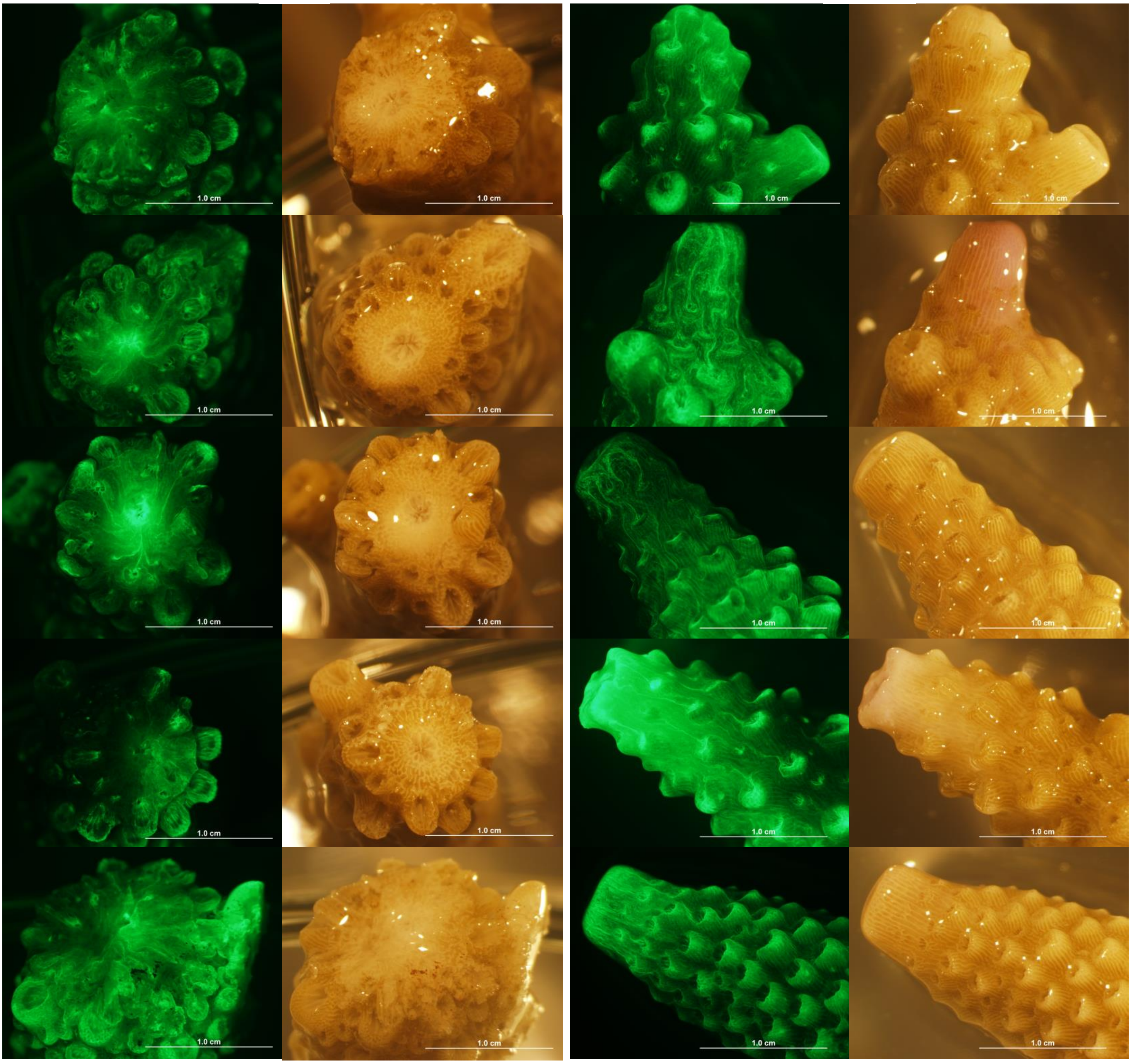

Figure 4. Stereoscopic microcopy images of cut (left) and non-cut (right) apical tips held under temperature-treated seawater conditions (~ 32ºC), day 4.

Day 6 Controls:

Cut **Uncut**

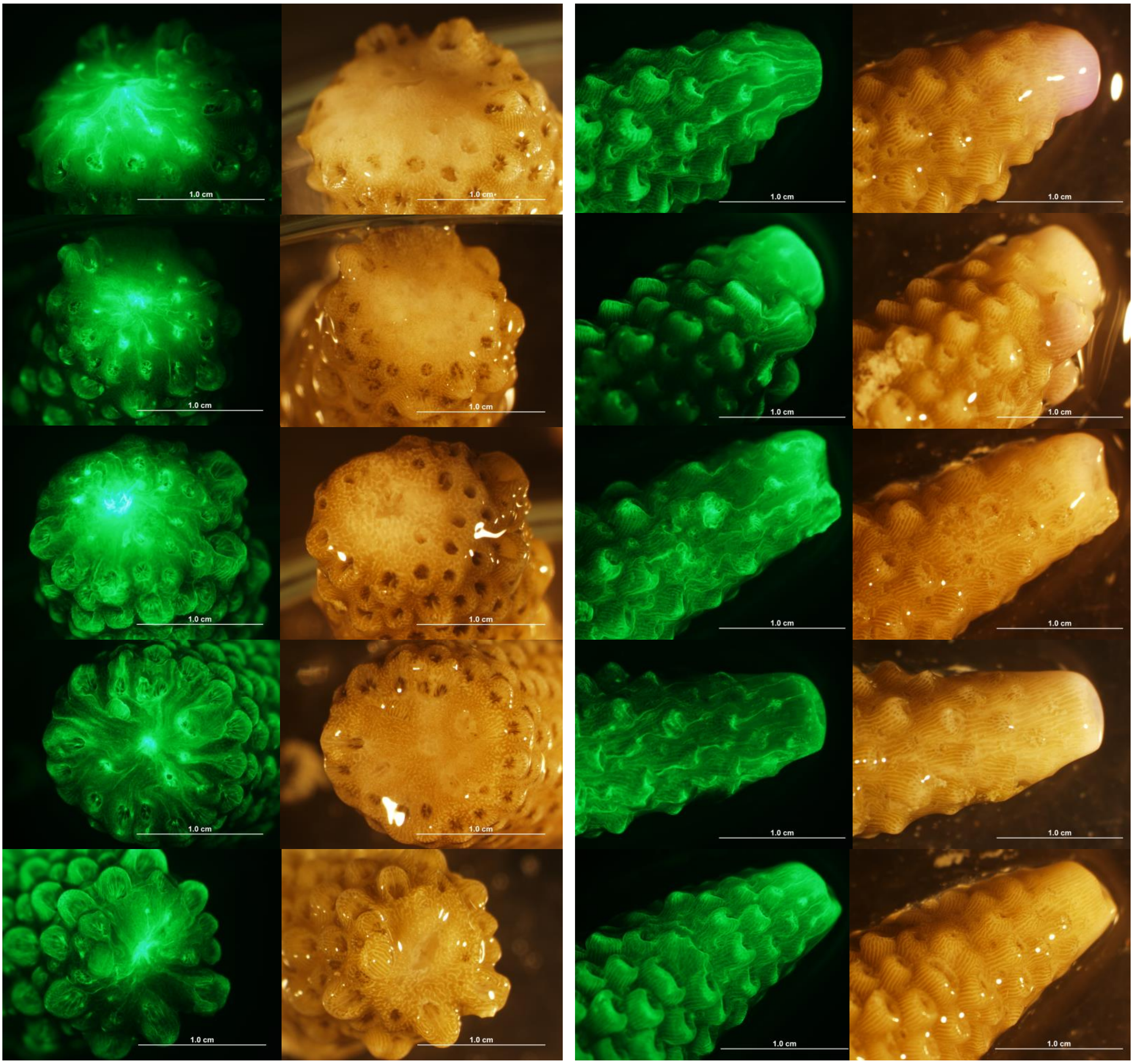

Figure 5. Stereoscopic microcopy images of cut (left) and non-cut (right) apical tips held under control seawater conditions (~ 25ºC), day 6.

Day 6 Treatments:

Cut **Uncut**

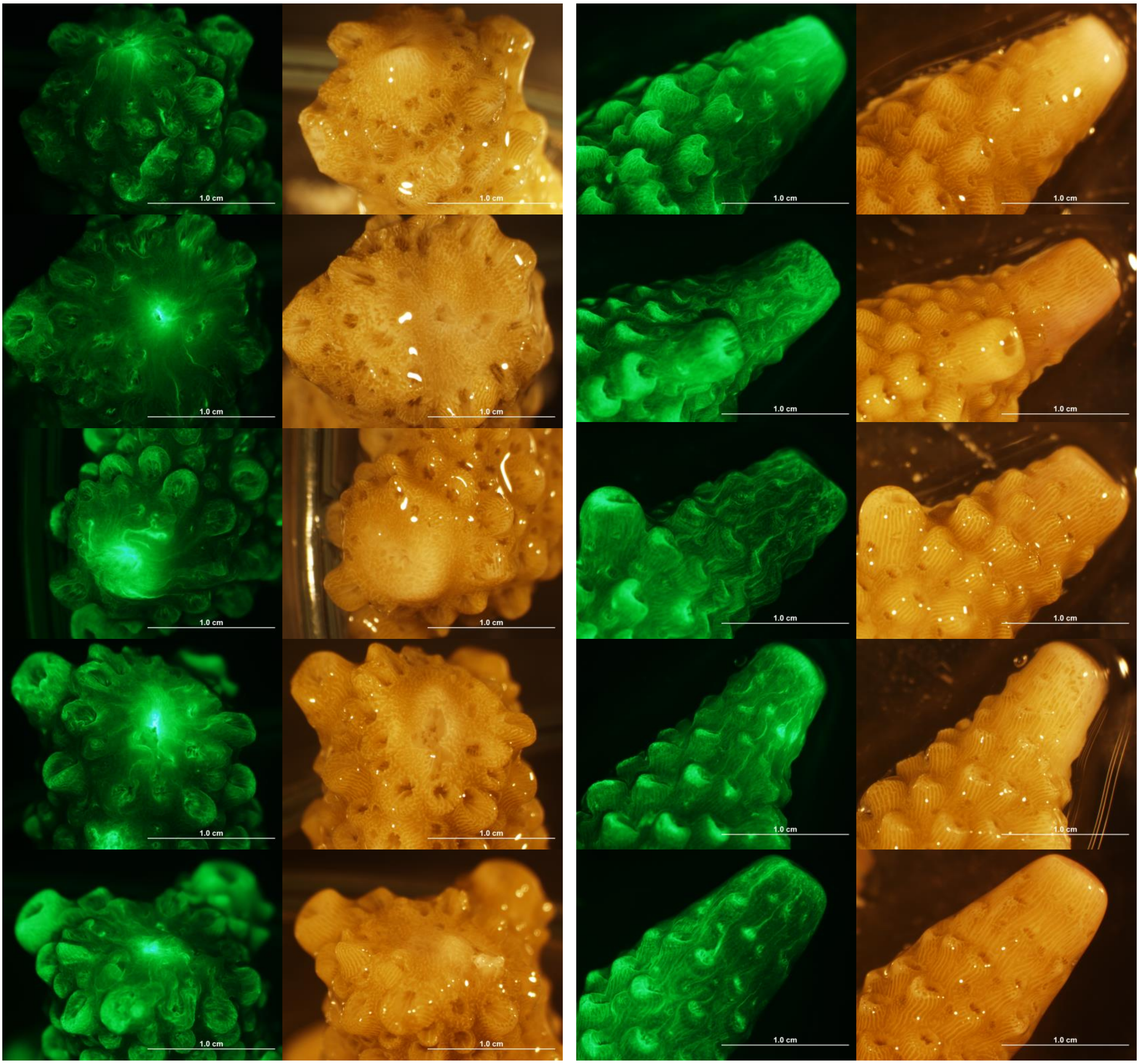

Figure 6. Stereoscopic microcopy images of cut (left) and non-cut (right) apical tips held under temperature-treated seawater conditions (~ 25ºC), day 6.

Day 8 Controls:

Cut | Uncut

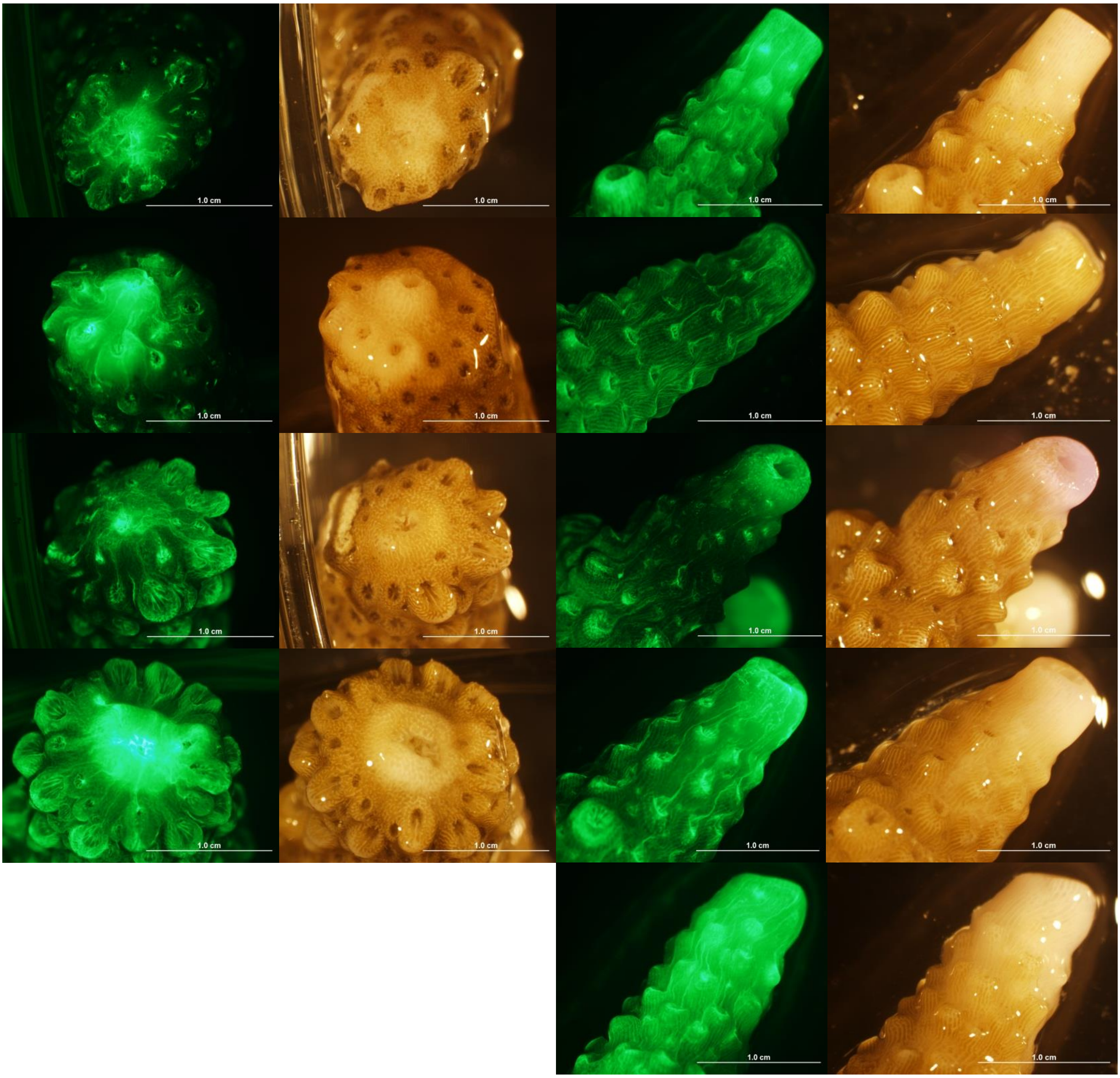

Figure 7. Stereoscopic microcopy images of cut (left) and non-cut (right) apical tips held under control seawater conditions (~ 25ºC), day 8.

<u>Day 8 Treatments:</u>

Cut **Uncut**

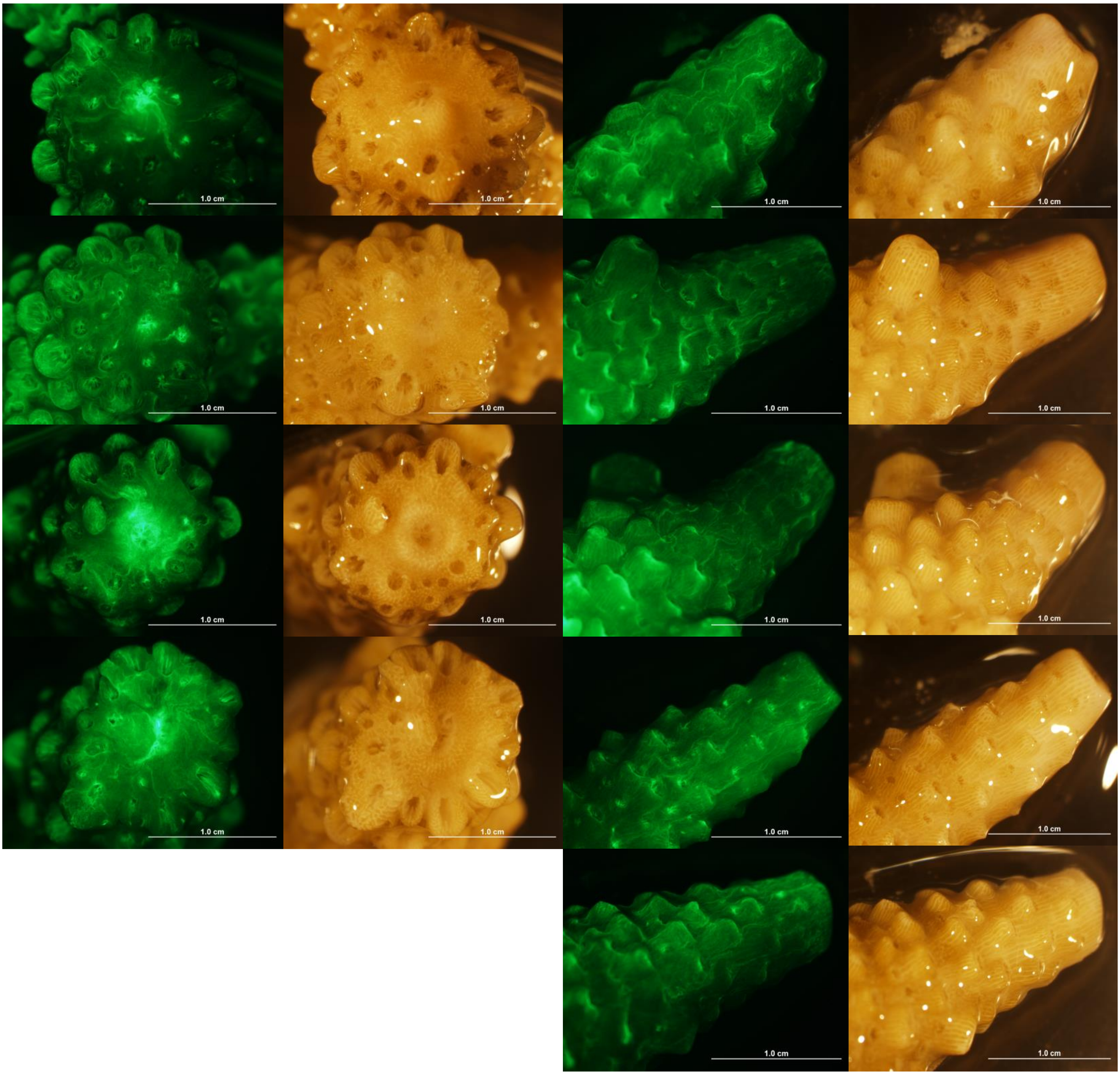

Figure 8. Stereoscopic microcopy images of cut (left) and non-cut (right) apical tips held under temperature-treated seawater conditions (~ 32ºC), day 8.

Day 10 Controls:

Cut **Uncut**

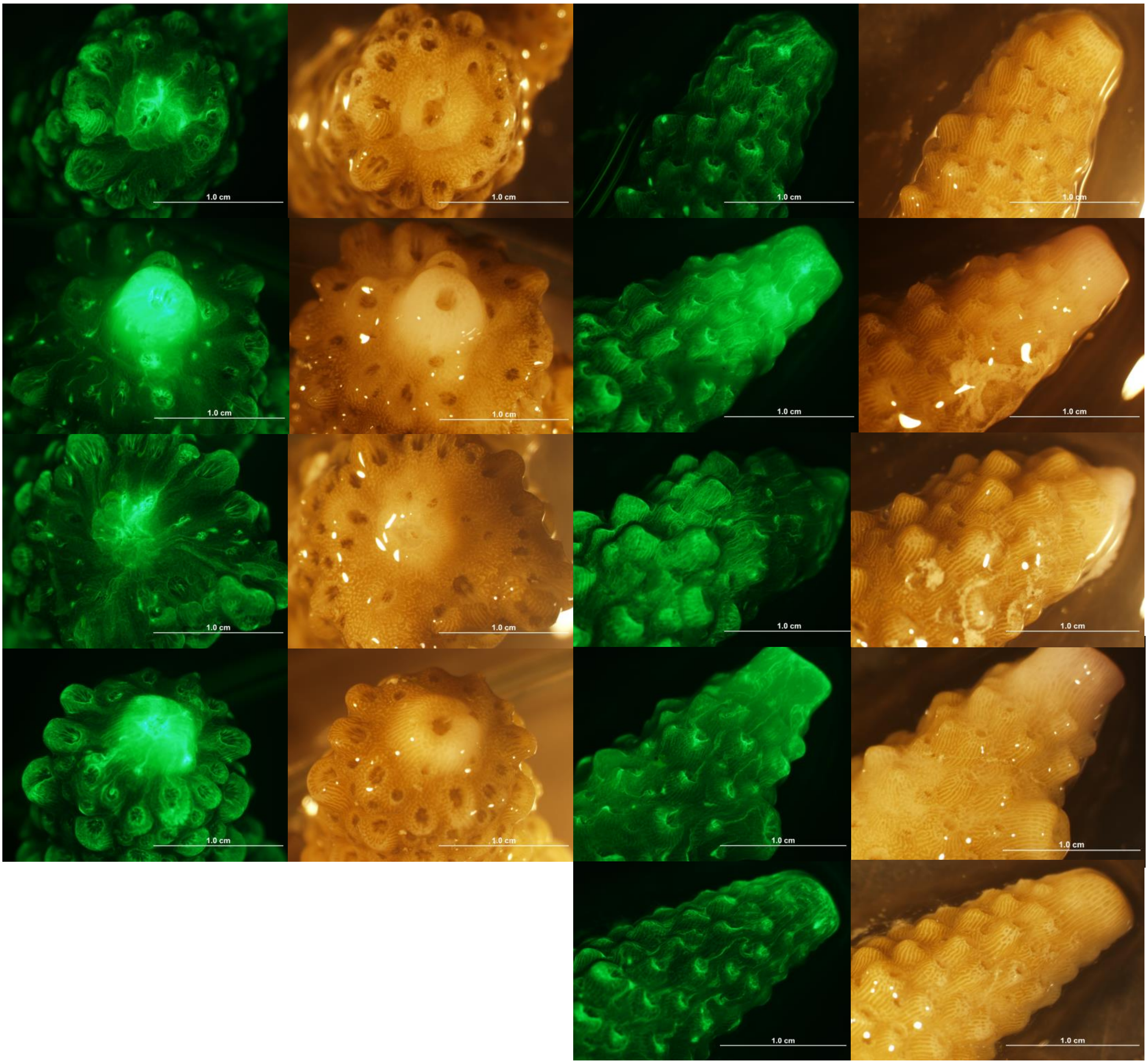

Figure 9. Stereoscopic microcopy images of cut (left) and non-cut (right) apical tips held under control seawater conditions (~ 25ºC), day 10.

Day 10 Treatments:

Cut **Uncut**

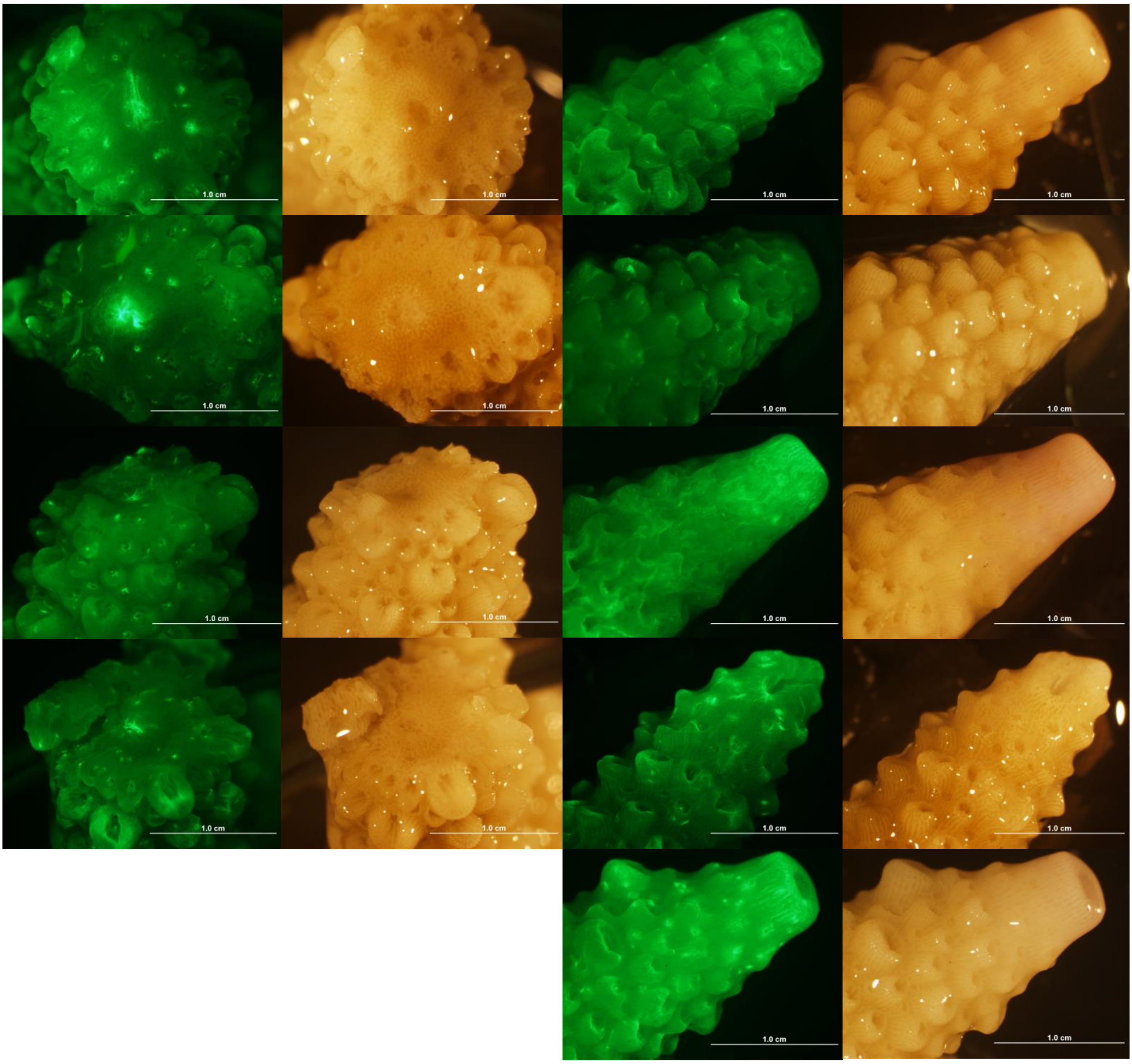

Figure 10. Stereoscopic microcopy images of cut (left) and non-cut (right) apical tips held under temperature-treated seawater conditions (~ 32ºC), day 10.

Day 12 Controls:

Cut **Uncut**

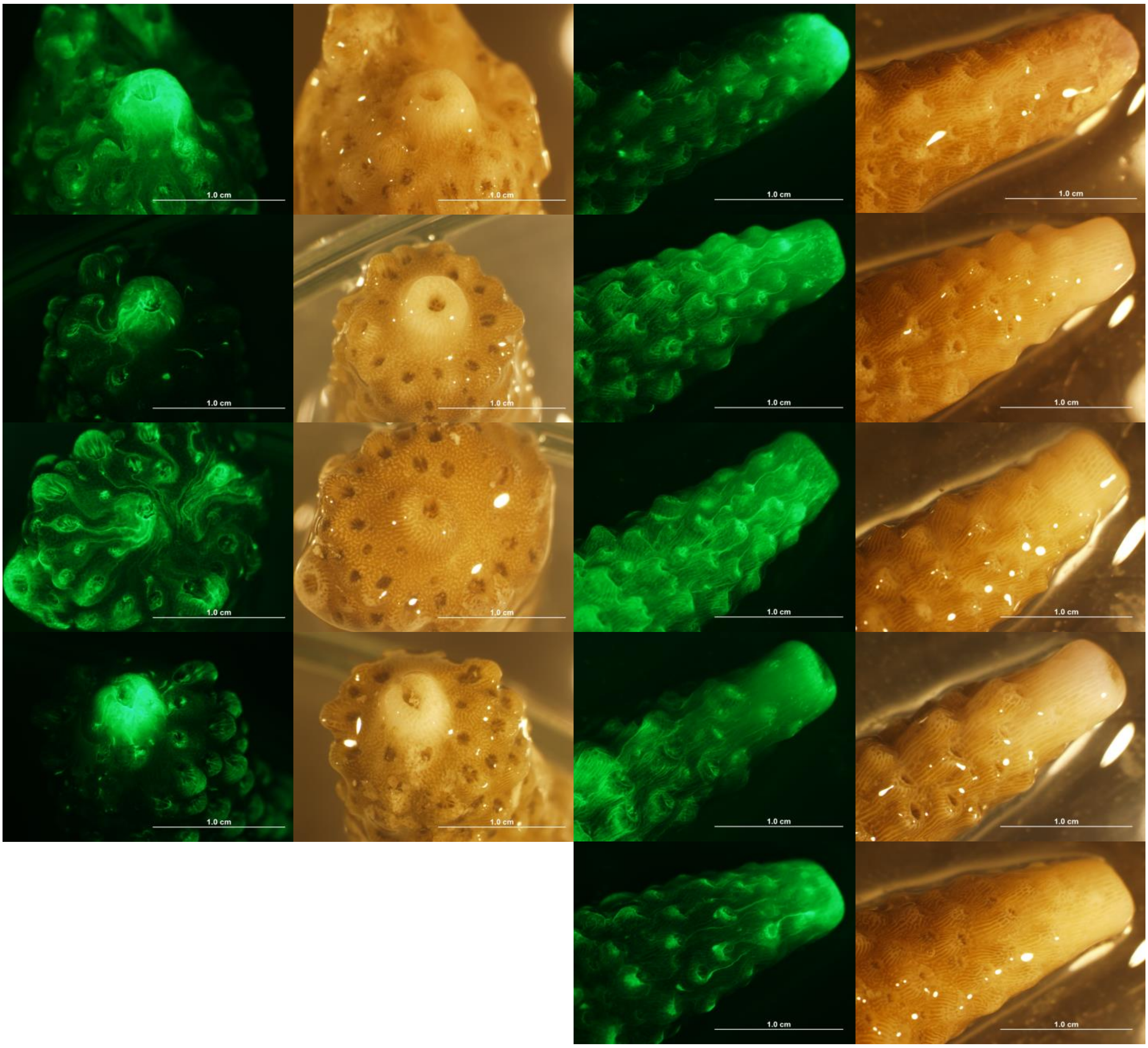

Figure 11. Stereoscopic microcopy images of cut (left) and non-cut (right) apical tips held under control seawater conditions (~ 25ºC), day 12.

Day 12 Treatments:

Cut **Uncut**

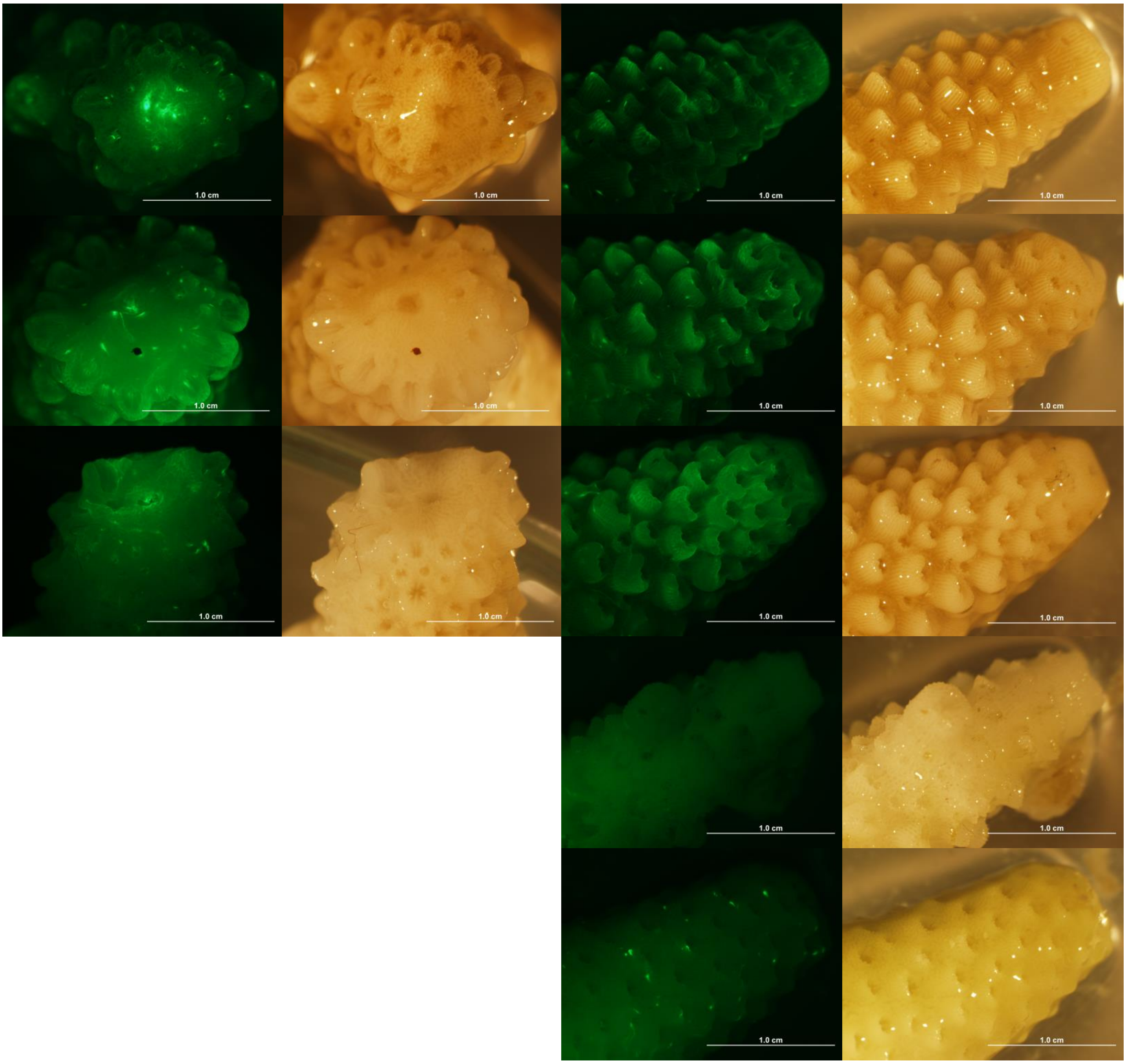

Figure 12. Stereoscopic microcopy images of cut (left) and non-cut (right) apical tips held under temperature-treated seawater conditions (~ 32ºC), day 12.

SEM SUPLEMENTARY MICROGRAPHS

Control cut (Days 2- 12)

Temperature cut (Days 2 -12)

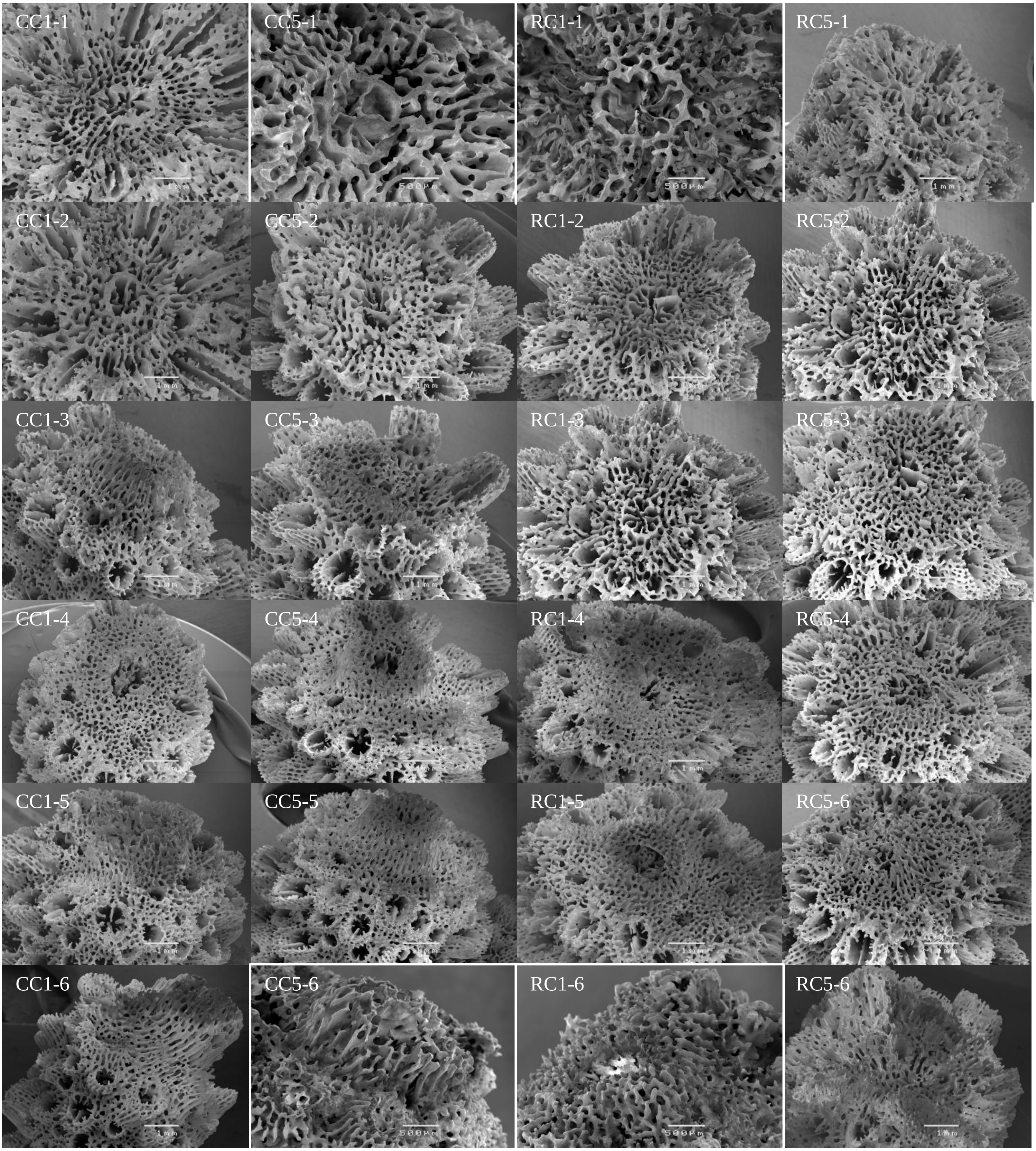

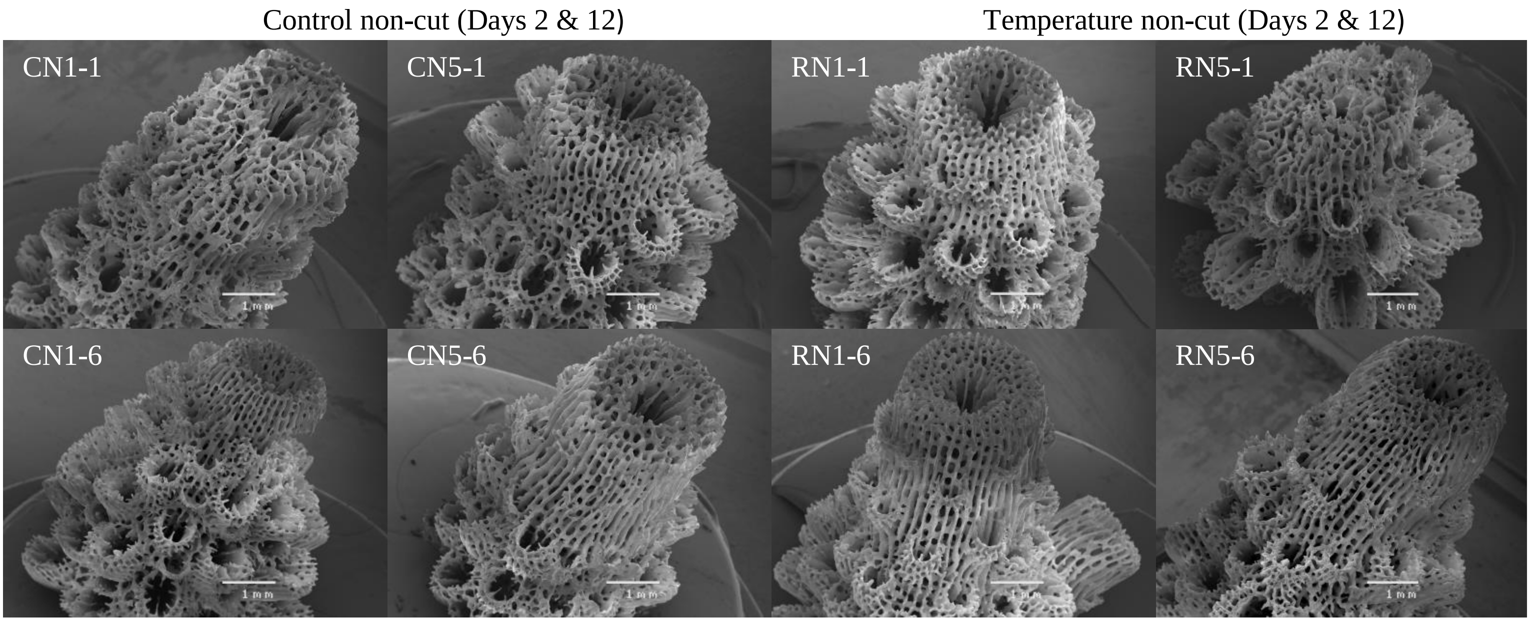

Figure 14. Supplementary SEM micrographs. Above shows the total samples imaged under SEM for the purpose for the qualitative study of skeletal regeneration of the apical tip.
